# Supplementary material for: Targeting integrin pathways: mechanisms and advances in therapy
Source: Signal Transduct Target Ther. 2023 Jan 2;8:1. doi: 10.1038/s41392-022-01259-6 (PMC9805914; doi:10.1038/s41392-022-01259-6)
Supplement: Supplementary file 1 — Similarity Report [file 41392_2022_1259_MOESM1_ESM.pdf]

PAPER NAME

13472989_YiminCui_Targetingintegrinpathwaysmechanismsandadvancesintherapy.docx

WORD COUNT

20014 Words

CHARACTER COUNT

120971 Characters

PAGE COUNT

36 Pages

FILE SIZE

932.0KB

SUBMISSION DATE

Nov 13, 2022 9:08 PM GMT+8

REPORT DATE

Nov 13, 2022 9:10 PM GMT+8

24% Overall Similarity

The combined total of all matches, including overlapping sources, for each database.

- 12% Internet database
- 22% Publications database
- Crossref database
- Crossref Posted Content database
- 0% Submitted Works database

Excluded from Similarity Report

- Bibliographic material
- Quoted material

Review Article

Targeting integrin pathways: mechanisms and advances in therapy

Xiaocong Pang[1,2#], Xu He[1,2#], Zhiwei Qiu[1,2#], Hanxu Zhang[1,2#], Ran Xie[1,2], Zhiyan Liu[1,2], Yanlun Gu[1,2], Nan Zhao[1,2], Qian Xiang[1,2]*, Yimin Cui[1,2]*

[1] Institute of Clinical Pharmacology, Peking University, Xueyuan Road 38, Haidian District, 100191 Beijing, China

[2] Department of Pharmacy, Peking University First Hospital, Xishiku Street, Xicheng District, 100034 Beijing, China

Correspondence and requests for materials should be addressed to Yimin Cui (e-mail: cui.pharm@pkufh.com) and Qian Xiang (e-mail: xiangqz@126.com).

ABSTRACT

Integrins are considered the main cell adhesion transmembrane receptors that play multifaceted roles as extracellular matrix (ECM)-cytoskeletal linkers and transducers in biochemical and mechanical signals between cells and their environment in a wide range of states in health and diseases. Integrin functions are dependable on a delicate balance between active and inactive status via multiple mechanisms, including protein-protein interactions, conformational changes, and trafficking. Due to their exposure on the cell surface and sensitivity to molecular blockade, integrins have been investigated as pharmacological targets for nearly 40 years, but given the complexity of integrins and sometimes opposite characteristics, targeting integrin therapeutics has been a challenge. To date, only seven drugs targeting integrins have been successfully marketed, including abciximab, eptifibatide, tirofiban, natalizumab, vedolizumab, lifitegrast and carotegrast. Currently, there are approximately 90 kinds of integrin-based therapeutic drugs or imaging agents in clinical studies, including small molecules, antibodies, synthetic mimic peptides, antibody-drug conjugates (ADCs), chimeric antigen receptor (CAR) T-cell therapy, imaging agents, etc. A serious lesson from past integrin drug discovery and research efforts is that successes rely on both a deep understanding of integrin regulatory mechanisms and unmet clinical needs. Herein, we provide a systematic and complete review of all integrin family members and integrin-mediated downstream signal transduction to highlight ongoing efforts to develop new therapies/diagnoses from bench to clinic. In addition, we further discuss the trend of drug development, how to improve the success rate of clinical trials targeting integrin therapies, and the key points for clinical research, basic research and translational research.

INTRODUCTION

Integrins have emerged as cell adhesion transmembrane receptors that serve as extracellular matrix (ECM)-cytoskeletal linkers and transduce biochemical and mechanical signals between cells and their environment in a wide range of states in health and diseases since their discovery in the 1980s [1-3] (Fig. 1). In mammals, each integrin heterodimer comprises an α-subunit and a β-subunit in a noncovalent complex, and 18 α- and 8 β-subunits create 24 functionally distinct heterodimeric transmembrane receptors [4]. Each α or β subunit contains a large ectodomain, a single-span helical transmembrane domain, and a short cytosolic tail, with the exception of β4 [5]. The majority of integrin heterodimers contain the β1 subunit and αv subunit. The β1 subunit can form heterodimeric complexes with 12 α-subunits, but β4, β5, β6, and β8 only interact with one α-subunit. Most α-subunits only form one kind of complex with one β partner, while α4 and αv interact with more than one β partner, including α4β1, α4β7 and αvβ1, αvβ3, αvβ5, αvβ6, αvβ8.

The "integrin" terminology originates from its function as the integral membrane protein complex bridging the ECM to the cytoskeleton [6]. The first integrins discovered were isolated based on their binding ability to fibronectin [1]. Typically, integrins can interact with a plethora of ECM proteins, and most of them contain small peptide sequences as integrin recognition motifs [7,8]. The targeting integrin sequences can be as simple as the Arg-Gly-Asp (RGD) or Leu-Asp-Val (LDV) tripeptides or more complex as GFOGER peptide [9-11]. According to the different binding characteristics of integrins, integrins can be divided into four types: leukocyte cell adhesion integrins, RGD-binding integrins, collagen (GFOGER)-binding integrins, and laminin-binding integrins [12]. Classically, there are 8 members in the RGD-binding family of integrins: αvβ1, αvβ3, αvβ5, αvβ6, αvβ8, α8β1, α5β1, and αIIbβ3. The RGD peptide is the common binding motif of these RGD-binding integrins in the ECM (e.g., fibronectin, osteopontin, vitronectin, and fibrinogen) [13].

Leukocyte cell adhesion integrins consist of 8 members, including α4β1, α9β1, αLβ2, αMβ2, αXβ2, αDβ2, α4β7, and αEβ7. Integrins α4β1, α4β7, α9β1 and αEβ7 also recognize short specific LDV peptide sequences, and an LDV motif is also present in fibronectin. β2 is the most common integrin that mediates leukocyte adhesion and migration, which is characterized by sites within ligands that are structurally similar to the LDV motif [14]. The four collagen-binding integrins (α1β1, α2β1, α10β1, and α11β1) recognize the triple helical GFOGER sequence in the major collagens, but their binding ability *in vivo* depends on the fibrillar status and the accessibility of interactive domains [12]. Four non-α I domain-containing laminin-binding integrins (α3β1, α6β1, α7β1, and α6β4) can bind with laminins. In addition, three α I domain-containing integrins (α10β1, α2β1, and α1β1) can form a distinct laminin/collagen-binding subfamily. The expression of these integrin isoforms is tissue specific and developmentally regulated; however, a full understanding of their role is still lacking. Beyond classical ECM mediators, integrins are also reported to interact with a diversity of non-ECM proteins on the surfaces of prokaryotic, eukaryotic, and fungal cells, as well as a range of viruses [15,16]. In addition, integrins can also be exploited as cell surface receptors for growth factors, hormones and polyphenols [17].

The wide range of ECM and non-ECM molecules makes integrins integral mediators of cell biology in mass. Integrin functions are dependable on a delicate balance between active and inactive status via multiple mechanisms, including protein–protein interactions, conformational changes, and trafficking [4]. These processes are triggered through "inside-out" signals and "outside-in" signals, resulting either from interacting with proteins such as α-actinin, talin, vinculin, and paxillin to the cytoplasmic β-integrin tail or from binding to ECM ligands and recruiting adhesion complexes [18,19]. Upon adhesion, cytoskeletal proteins are linked to the integrin β-subunit cytoplasmic tail [20]. Most integrin adhesion complexes (IACs) include focal adhesions (FAs), fibrillar adhesions, immunological synapses, and podosomes [21]. The primary intracellular downstream signaling mediators of integrins refer to focal adhesion kinase (FAK), Src-family protein tyrosine kinases, and integrin-linked kinase (ILK) [22]. Integrins transduce mechanical and biochemical signals to promote cell proliferation, adhesion, spreading, survival and ECM assembly and remodelling.

Due to their exposure on the cell surface and sensitivity to molecular blockade, integrins have been investigated as pharmacological targets for nearly 40 years, and a certain amount of current efforts involving integrin therapeutics continues to surprise (Fig. 1). In 2022, the Lasker Prize in Medicine was awarded to Richard Hynes, Erkki Ruoslahti, and Timothy Springer for groundbreaking research in the discovery of integrins, which aroused great concern about the field of integrins. The integrin discovery history started in the 1980s. The first identification of integrin family member is αIIbβ3, and the first integrin-targeting drug was Abciximab, approved in 1994 as an αIIbβ3 antagonist [23]. Intravenous αIIbβ3 inhibition has been a major success in the treatment of coronary artery disease, but current oral αIIbβ3 antagonists have failed to achieve end points but potentially induce a direct toxic effect with prothrombotic mechanisms [24]. In 2003, a nanotherapeutic agent, a nanoparticle coupled to an αvβ3-targeting ligand for delivering genes, was first reported to selectively target angiogenic blood vessels in tumor-bearing mice [25]. In 2003, the αL antagonist Efalizumab was approved but withdrawn in 2009 due to the adverse effect of progressive multifocal leukoencephalopathy. In 2004, the pan-α4 antagonist natalizumab was approved for multiple sclerosis. Then, there is a real gap in the market for targeting integrins. The failure of cilengitide in clinical trials on glioblastoma treatment had a huge impact on targeting αv-integrin drug discovery [26]. To date, there are no approved drugs targeting αv-integrin. In 2014 and

2016, vedolizumab and lifitegrast, targeting $\alpha 4\beta 7$ and $\alpha L\beta 2$ for the treatment of inflammatory bowel disease and dry eye disease, respectively, were approved. In 2017, CAR T cells targeting integrin were investigated [27]. In 2022, there will be a large breakthrough targeting integrin, including the phase III clinical trial success of the 99mTc-3PRGD2 imaging agent, the approval of Carotegrast, as the first oral anti-integrin drug, by Japan's Pharmaceuticals and Medical Devices Agency (PMDA), and the phase IIa positive results of the oral $\alpha v\beta 6/\alpha v\beta 1$ antagonist PLN-74809. To date, the U.S. Food and Drug Administration (FDA) has approved a total of 7 drugs targeting integrins [28]. Currently, there are approximately 90 kinds of integrin-targeting therapies in clinical trials, including integrin antagonists and imaging agents, including small molecules, antibodies, synthetic mimic peptides, antibody-drug conjugates (ADCs), CAR T-cell therapy, imaging agents, etc. A serious lesson from past integrin drug discovery and research efforts is that successes rely on both a deep understanding of integrin regulatory mechanisms and unmet clinical needs.

Several recent reviews have analyzed the details of both biochemical and mechanical integrin regulation, integrin structure, integrin roles in cancer and fibrosis disease, RGD-binding integrin drug discovery, especially small-molecule inhibitors of the αv integrins, the mechanism of endocytosis, exocytosis, intracellular trafficking, and mechanotransduction [3,4,28,29]. Herein, we attempt to provide a systematic and complete review of all integrin family members and integrin-mediated downstream signal transduction to highlight ongoing efforts to develop new therapies/diagnoses. Furthermore, we also provide insight into the trend of drug development, how to improve the success rate of clinical trials of integrin-targeting therapies, and the key points for clinical research, basic research and translational research.

STRUCTURE AND FUNCTION OF THE INTEGRIN FAMILY

Since the crystal structure of $\alpha v\beta 3$ was available in 2001, conformational changes in integrin ectodomains have been illustrated. The ectodomain of an α-subunit contains four extracellular domains: a seven-bladed β-propeller, a thigh, and two calf domains (Fig. 2 a, b). The common structure of different α-subunits present in their extracellular domain are seven repeat motifs, which fold into a seven-bladed propeller structure on the upper surface, and on the lower surface of blades 4–7, divalent cation-binding sites are located (Fig. 2 a, b). Half of the integrin α subunits (i.e., $\alpha 1$, $\alpha 2$, $\alpha 10$, $\alpha 11$, αD, αX, αM, αL) contain a domain of 200 amino acids, known as the inserted (I) domain or αA domain, which is located between blades 2 and 3 of the β propeller. Integrins with an α I domain bind to ligands through this domain [30]. The structure of an α I domain contains a metal ion-dependent adhesion site motif (MIDAS), which is the major ligand-binding site [31].

The crystal structure of the α I domain suggests three distinct conformations, termed bent closed, extended closed and extended open conformations [32] (Fig. 2 c). They differ not only in the coordination of the metal in the MIDAS but also in the arrangement of the βF-α7 (F/α7) and the disposition of the α1 and α7 helices [32,33]. In the active state of the α I domain, a C-terminal glutamate from the α I domain ligates the β I MIDAS and further stabilizes the high-affinity conformations [34]. The ectodomain of the β subunit comprises seven domains with complex domain insertions (Fig. 2 a, b): a β I domain with insertion in the hybrid domain, plexin-semaphorin-integrin (PSI), four cysteine-rich epidermal growth factor (EGF) modules, and a beta tail domain (βTD) domain [35]. The integrin β subunit I domain is homologous to the α I domain. Resting integrins exist in a bent-closed conformation, which is unable to bind ligand, and Integrins can extend and form a high-affinity conformation with an open headpiece [36,37]. The open headpiece conformation is induced with binding ligands, and this activated state possesses high binding affinity. Ligand binding further

provides the energy for conformational change triggering outside-in signaling. In addition, for induction of the high-affinity state, the open headpiece conformation could be produced artificially by mutations [38]. For example, it was reported that mutations in βTD residues in CD11b/CD18 integrins could lead to constitutive activation and outside-in signaling responses [35].

All α I domain less integrins bind to the ligand directly using a binding pocket that is formed by the β-propeller/β I domain interface [21]. In this ligand binding pocket, three divalent metal ion binding sites are concentrated on the ligand binding sites of the β I domain in a linear arrangement [39]. The middle site, like the α I domain, called MIDAS, whose metal ion directly coordinates the side chain of the acidic residue characteristic of the integrin ligands, and the two outer sites, adjacent metal ion-dependent binding site (ADMIDAS) and ligand-associated metal binding site (LIMBS) or synergistic metal ion-binding site (SyMBS) [40,41], can also bind Mn^{2+}, Mg^{2+} and Ca^{2+}, sharing some coordinating residues in common with MIDAS [42-44]. The divalent metal cation on MIDAS is essential for the binding of integrin ligands. Some studies have shown that after the metal ions in MIDAS are removed by residue mutations, the ligand fails to bind to integrins, which suggests that MIDAS is critical for coordination and binding [43].

The first crystal structure of αvβ3 bound to a mutant of fibronectin revealed the structure basis underlying pure antagonism, a central π - π interaction between Trp1496 in the RGD-containing loop of the high affinity form of the 10th type III RGD-domain of fibronectin (FN) (hFN10) and Tyr122 of the β3-subunit that blocked conformational changes triggered by a wild-type form (wtFN10) and trapped hFN10-bound αvβ3 in an inactive conformation [45]. Then, the cyclic peptide CisoDGRC and small-molecule antagonists of αIIbβ3 and αvβ3 were reported to retain high affinity without apparently inducing the conformational change in αvβ3 by the same mechanism, interacting with β3 Tyr122 on the β1-α1 loops and preventing its movement toward MIDAS, which is a key element in triggering conformational change [46-48]. Recently, Lin et al. [49] proposed that the water molecule between the Mg^{2+} ion and the MIDAS serine side chain is also important for the integrin conformational change, and expulsion of this water is a requisite for the transition to the open conformation. Therefore, direct evidence for distinct functional roles for conformational change is still acquired for integrin-targeting drug development.

RGD-binding integrins

RGD-binding integrins refer to a class of integrins that bind with the tripeptide motif Arg-Gly-Asp in ECM proteins, including αvβ1, αvβ3, αvβ5, αvβ6, αvβ8, α5β1, α8β1, and αIIbβ3 [50,51] (Fig. 3).

Integrin αvβ1 primarily binds with transforming growth factor-β (TGF-β), fibronectin, osteopontin, and neural cell adhesion molecule L1 [52]. In fibroblasts, such as hepatic stellate cells and pulmonary fibroblasts, integrin αvβ1-induced TGF-β activation is important in ECM accumulation [53,54]. It also mediates the adhesion of osteoblasts to connective tissue growth factor, which induces cytoskeleton reorganization and cell differentiation [55]. Recently, integrin αvβ1 was identified as a regulator that mediates the vascular response to mechanical stimulation [56].

Integrin αvβ3 is one of the earliest integrins to be studied. Because of its specific binding with vitronectin, integrin αvβ3 was originally called the vitronectin receptor. However, further studies found that integrin αvβ3 also binds with many other ligands, such as TGF-β, fibronectin, osteopontin, neural cell adhesion molecule L1, fibrinogen, von Willebrand factor, thrombospondin, fibrillin, and tenascin [52]. It is widely expressed in mesenchyme and blood vessels, smooth muscle cells, fibroblasts, and platelets [57]. Integrin αvβ3 participates in angiogenesis, ECM regulation, vascular

smooth muscle cell migration, and osteoclast adhesion to the bone matrix [57]. In addition, integrin αvβ3 expressed in leucocytes participates in regulating monocyte, macrophage, and neutrophil migration and dendritic cell and macrophage phagocytosis, which regulates inflammation progression [58,59].

Integrin αvβ5 binds with TGF-β, osteopontin, vitronectin, bone sialic protein, thrombospondin, and nephroblastoma overexpressed (NOV, also known as CCN3) [52]. Integrin αvβ5-induced TGF-β activation is involved in various physiological processes, such as wound healing mediated by myofibroblasts [60], matrix molecule synthesis by airway smooth muscle [61], and type I procollagen expression in skin fibroblasts [62]. The binding of integrin αvβ5 with vitronectin is essential for cerebellar granule cell precursor differentiation by regulating axon formation [63]. In addition, integrin αvβ5 is highly expressed in mature intestinal macrophages and mediates macrophage phagocytosis of apoptotic cells [64,65].

Integrin αvβ6 primarily binds with TGF-β, fibronectin, osteopontin, and a disintegrin and metalloproteinase (ADAM) [52,66]. It is an important activator of TGF-β, which regulates innate immunity and anti-inflammatory surveillance in the lungs, junctional epithelium of the gingiva, skin, and gastrointestinal tract [67-69]. In addition, it participates in the process of tooth enamel formation[68]. Studies have reported that β6 subunit of αvβ6 integrin (ITGB6) knockout significantly increases the risk of emphysema [70], causes hypomineralized amelogenesis imperfecta [71], promotes skin inflammation and hyperplasia [68], and accelerates skin wound repair [72].

Integrin αvβ8 is a receptor for TGF-β, which activates TGF-β signal transduction by binding with TGF-β [73]. Integrin αvβ8-mediated TGF-β activation is involved in regulating neurovascular development, immune cell recruitment and activation, and stem cell migration or differentiation (such as neuroblast chain and neural stem cell migration, nonmyelinating Schwann cell and mesenchymal stem cell differentiation) [74].

Integrin α5β1 binds with numerous ligands, such as fibronectin, fibrinogen, fibrillin, osteopontin, and thrombospondin [75]. Owing to its diversity of ligands, integrin α5β1 is involved in numerous physiological processes, including promoting cell migration [76], invasion [77], proliferation [78], and aging [79]. The normal function of T cells is also inseparable from the participation of integrin α5β1, which affects the inflammatory process. In addition, integrin α5β1 is adverse for the formation of bone tissue, and upregulation of integrin α5β1 causes the loss of bone tissue-forming capacity in adipose-derived stromal/stem cells [80].

Integrin α8β1 binds with TGF-β, tenascin, fibronectin, osteopontin, vitronectin, and nephronectin [52]. It is highly expressed in contractile cells, such as vascular smooth muscle cells, neuronal cells, and mesangial cells [81]. Integrin α8β1 functions as a cell migration regulator that promotes or inhibits cell migration according to the differentiated state of cells [81]. It promotes the migration of cells that are not initially contractile (such as mesangial cells, vascular smooth muscle cells, and hepatic stellate cells) and inhibits the migration of cells that are differentiated for contractile function (such as neural cells) [81,82].

Integrin αIIbβ3 is primarily expressed in platelets and their progenitors [83]. It binds with fibrinogen, fibronectin, thrombospondin, vitronectin, von Willebrand factor, and so on [52]. Integrin αIIbβ3 plays a central role in maintaining platelet adhesion, spreading, aggregation, clot retraction, and thrombus consolidation, resulting in platelet activation and arterial thrombosis [84].

Leukocyte cell adhesion integrins

Leukocytes constitutively express several types of integrins, including α4β1, α9β1, αLβ2,

αMβ2, αXβ2, αDβ2, α4β7, and αEβ7 [85](Fig. 3). Among them, integrins containing the β2 subunit are most abundant in leukocytes; therefore, integrin β2 is also called a leukocyte integrin [86].

Leukocyte cell adhesion integrins are primarily involved in the regulation of inflammation. When infection occurs, leukocytes, such as neutrophils, eosinophils, and basophils, are carried close to the site of infection by blood flow [87,88]. Selectins expressed on leukocytes then bind with their ligands on vascular endothelial cells, which makes leukocytes adhere to the vascular endothelium and start fast rolling [86]. This process provides enough time for integrins to bind with their ligands. Integrins αLβ2 (bound to intercellular adhesion molecule [ICAM]-1), αMβ2 (bound to ICAM-2) and α4β1 (bound to vascular cell adhesion molecule [VCAM]-1) are activated, slowing the rolling of leukocytes [86]. As leukocytes stop in the vascular endothelium, active integrin αLβ2 and αMβ2 induce leukocyte spreading and crawling toward infiction[89]. Leukocytes that reach the site of infection cross the vascular endothelium and enter infected tissue with the participation of integrin α6β1, thereby mediating the inflammatory response [86,89].

In addition, integrin αLβ2 is also involved in enhancing the phagocytosis of bacteria by neutrophils [90]. It was reported that an integrin αLβ2 antibody effectively inhibited the phagocytosis of *Streptococcus* pyogenes by neutrophils [91]. Integrin αMβ2 was proven to be important in neutrophil phagocytosis, reactive oxygen species (ROS) formation, neutrophil extracellular traps (NETs), apoptosis, and cytokine production, thereby regulating inflammation and defending against microbial infection [90]. Integrins αXβ2 and αMβ2 are homologous adhesion receptors that are expressed on similar types of leukocytes and share many receptors [92]. It plays a central role in regulating the anti-inflammatory function of macrophages [92]. Deficiency of integrin αXβ2 results in the loss of antifungal activity of macrophages by eliminating its recruitment and adhesion function [92] and disturbs dendritic cell recruitment to the infection site [93]. Integrin αDβ2 is highly homologous to integrin αMβ2 and αXβ2. It binds with ICAM-1, ICAM-3, and VCAM-1, thereby playing an important role in regulating inflammation and microbial infection [90,94].

Integrin αEβ7 is mainly expressed in lymphocytes of intestinal, lung and skin epithelial tissues as well as in conventional dendritic cells of mucosa and dermis [95]. The interaction between integrin αEβ7 and E-cadherin mediates lymphocyte attachment to intestinal and skin epithelial cells [95]. In human hematopoietic stem cells and progenitor cells, integrin α1β9 regulates cell adhesion and differentiation in the endosteal stem cell niche, thereby regulating hematopoietic processes [96]. In addition, integrin α1β9 is also involved in the regulation of cell adhesion and migration in numerous organs, such as the skin, liver, and spleen [97]. Integrin α4β7 specifically binds VCAM-1 and mucosal address in cell-adhesion molecule-1 (MAdCAM-1) to regulate lymphocyte migration, which mediates the homing of lymphocytes to gut tissues [98,99].

Collagen (GFOGER)-binding integrins

Collagen-binding integrins refer to a class of integrins that bind with GFOGER-like sequences in collagen, including α1β1, α2β1, α10β1, and α11β1 [100] (Fig. 3).

Integrin α1β1 was first identified in activated T cells [101]. It is also expressed in connective tissue cells (such as mesenchymal stem cells and chondrocytes) and cells that are in contact with basement membranes (such as smooth muscle cells, pericytes, and endothelial cells) [102]. Integrin α1β1 binds with collagens I, III, IV, IX, XIII, XVI and collagen IV chain-derived peptide arrest [102,103]. In leukocytes, integrin α1β1 functions as a promoter of T cells in inflammatory responses [104,105] and mediates monocyte transmigration by binding with collagen XIII [106]. In bone, integrin α1β1 plays an important role in damage repair processes. It has been reported that knockout of

integrin β1 (ITGB1) results in slowed proliferation of mesenchymal stem cells and inhibition of cartilage production, thereby hindering fracture healing and promoting osteoarthritis [107,108].

Integrin α2β1 is expressed in fibroblasts, T cells, myeloid cells, megakaryocytes, platelets, keratinocytes, epithelial cells, and endothelial cells [100,109]. Integrin α2β1 binds with collagens I, III, IV, V, XI, XVI and XXIII [109]. It also binds with lumican and decorin, which are proteoglycans [110,111]. In platelets, integrin α2β1 participates in stabilizing thrombi by binding with collagen I [112,113]. In T helper cell 17, integrin α2β1 cooperates with interleukin 7 receptor to mediate bone loss [114].

Integrin α10β1 is expressed in fibroblasts, chondrocytes, chondrogenic mesenchymal stem cells and cells lining the endosteum and periosteum [115]. It primarily binds with collagens II and is essential in cartilage production and skeletal development [115,116]. Integrin α10β1 is regarded as a biomarker of chondrogenic stem cells [115]. A previous study revealed that integrin α10β1 deficiency resulted in cartilage defects and chondrodysplasia [117].

Integrin α11β1 is expressed in fibroblasts, mesenchymal stem cells, and odontoblasts [100,118]. It is important in tooth eruption, wound healing, and fibrosis [119,120]. The osteogenic differentiation of mesenchymal stem cells is driven by integrin α11β1 [121]. Studies have shown that integrin α11β1 deficiency results in incisor tooth eruption defects in mice [118]. In addition, integrin α11β1 also promotes myofibroblast differentiation, which accelerates dermal wound healing [113]. Knockout of integrin α11β1 reduced granulation tissue formation in mice [122].

Laminin binding integrins

Laminin binding integrins are a group of integrins that bind with laminins [123]. Laminins are macromolecular glycoproteins located in the ECM [124]. As the main component of the basement membrane, laminins play critical roles in regulating cell adhesion, proliferation, migration, and survival [125]. Laminins consist of various α, β, and γ subunits [126,127], which constitute 16 different laminin isoforms [126,127].

Integrins that have been identified as binding with laminins include α1β1, α2β1, α3β1, α6β1, α10β1, α6β4, α7β1, and αvβ3 [128-130] (Fig. 3). Integrins α1β1 and α2β1 bind with the N-terminal domain of laminin α1 and α2 chains [131-133]. Integrins α3β1, α6β1, α6β4, and α7β1 bind with the C-terminal domain of laminins [128,134]. Integrin αvβ3 binds with the L4 domain of the laminin α5 chain [129]. However, the physiological effects of the binding of α1β1, α2β1, α10β1, and αvβ3 with laminins are very limited, so we generally classify integrins α3β1, α6β1, α6β4, and α7β1 as laminin-binding integrins [134,135]. Integrins α1β1, α2β1, and α10β1 have been classified as collagen-binding integrins, and integrin αvβ3 has been classified as an RGD-binding integrin (as described above).

Integrin α3β1 is mainly expressed in the lung, stomach, intestine, kidney, bladder, and skin [125]. It mainly binds with laminin-332 and laminin-511 to mediate cell adhesion to the basement membrane and cell-to-cell communication [125]. Studies have found that integrin α3β1 plays a crucial role in the development of the brain, lung, liver, kidney, skin, muscle, and other organs [136-140]. Deficiency in integrin α3β1 causes symptoms such as skin blisters [141], disorganization of neurons in the cerebral cortex [142], fragmentation of the glomerular basement membrane[139], and death in neonatal mice within 24 hours of birth [139].

Integrin α6β1 is primarily expressed in platelets, leukocytes, gametes, and epithelial cells [125]. Laminin-111, laminin-511 and laminin-332 are the most highly affiliative ligands [143]. In the brain, integrin α6β1 may be involved in nervous system development [144]. In the ovary, the interaction of integrin α6β1 with laminins could inhibit progesterone production, thereby regulating luteal formation and follicle growth [145]. Moreover, integrin α6β1 in pericytes acts as a regulator of

angiogenesis by controlling the structure of platelet-derived growth factor (PDGF) receptor (PDGFR) β and the basement membrane [146].

Integrin α6β4 is expressed in subsets of endothelial cells, squamous epithelia, immature thymocytes, Schwann cells, and fibroblasts in the peripheral nervous system [147,148]. Both laminins and epidermal integral ligand proteins are ligands of integrin α6β4 [125]. Integrin α6β4 binds with laminins and mediates epithelial cell adhesion to the basement membrane, thus maintaining the integrity of epithelial cells [125]. In addition, integrin α6β4 binds with bullous pemphigoid (BP) antigen 1-e (BPAG1-e) and BP antigen 2 (BPAG2) to form hemidesmosomes (HDs), where the extracellular domain of integrin α6β4 binds with laminins and the intracellular domain of integrin α6β4 interacts with the actin cytoskeleton. This structure links the intracellular keratin cytoskeleton to the basement membrane and plays a critical role in regulating the stability of epithelial cell attachment [149-151]. In mice, integrin α6β4 deficiency results in reduced skin adhesion properties and extensive exfoliation of epidermal and other squamous cells, accompanied by loss of HDs on the basement membrane of keratinocytes [147,149]. These findings suggested that integrin α6β4 might be involved in epidermolysis bullosa [149,152]. In addition, integrin α6β4 is also involved in cell death, autophagy, angiogenesis, aging and differentiation regulation and plays a regulatory role in cancer, respiratory diseases, and neurological diseases [153,154].

Integrin α7β1 is mainly expressed in cardiac and skeletal muscles. It binds with laminin-211 and laminin-221 to mediate the binding of muscle fibers with myotendinous junctions. It has been found that integrin α7β1 deficiency may be one of the important causes of congenital myopathy [155], as integrin α7 (ITGA7) knockout mice develop muscular dystrophy [156]. In addition, integrin α7β1 participates in vascular development and integrity. Studies have revealed that integrin α7β1 deficiency causes abnormalities in the recruitment and survival of cerebral vascular smooth muscle cells, leading to vascular damage [157].

INTEGRIN-MEDIATED SIGNAL TRANSDUCTION

Inside-out signaling

Integrins act as adhesion and signaling receptors by bidirectionally transducing mechanotransduction and biochemical signals across the plasma membrane, which requires engagement of extracellular ligands by the integrin extracellular domains and recruits additional adaptor, cytoskeletal proteins and signaling molecules to their cytoplasmic tails [8,158]. The 3D structure of integrins determines their functional state. There are three basic conformations for integrin: a bent conformation, a medium-affinity conformation, and a high-affinity conformation [8,159] (Fig. 2c). Integrin activity corresponds to the integrin conformation: a bent conformation is associated with a ligand with low affinity, whereas a high affinity is associated with an extended conformation. In the bent conformation, both α and β subunits of the integrin are in a folded state, assuming a compact V-shaped conformation with the headpiece folded over the tailpiece, such that the ligand-binding site of the head is close to the proximal membrane end of both "legs". The affinity of integrin for extracellular ECM and integrin-mediated downstream events are regulated by the dynamic equilibrium between these conformations. The bent conformation is commonly maintained by endogenous inhibitory proteins. For example, shank-associated RH domain interacting protein (SHARPIN) in leukocytes and mammary-derived growth inhibitor (MDGI) suppress integrin activity by binding directly to the cytoplasmic tail of integrin α-subunit cytoplasmic tails [160,161]. Additionally, SHARPIN directly binds to integrin β1 cytoplasmic tails, and kindlin-1 can significantly enhance this interaction [162]. Integrin cytoplasmic associated protein-1 (ICAP1) acts as an inhibitor of β1 activation, which can

be antagonized by Krev/Rap1 Interaction Trapped-1 (KRIT1) [163]. Immunoglobin repeat 21 of filamin A (FLNa-Ig21) not only binds directly to the integrin β3 cytoplasmic tail but also interacts with the N-terminal helices of the αIIb and β3 cytoplasmic tails to stabilize the bent conformation [164].

In contrast, integrin-binding adaptor proteins inside the cell, including talins (talin 1 and talin 2), kindlins (kindlin 1, kindlin 2 and kindlin 3), vinculin, paxillin, FAK and others binding to the integrin cytoplasmic domain, trigger high-affinity extended integrin conformational changes. The extension of the extracellular domain, the separation of heterodimeric subunits from transmembrane parts in the membrane, and the rearrangement of the α β interface in the ligand binding domain release integrins from a compact bent conformation to an open conformation, and the ligand binding affinity increases. Then, integrins may cluster into many different types of adhesive complexes. This activation multi-step process is called activation or inside-out signaling [165], while the signal transmission direction of outside-in is the opposite [166] (Fig. 4). Talin is a main focal adhesion binding protein that initiates inside-out signaling by disrupting the interactions of the α and β subunits, known as the inner membrane clasp [167]. The head of talin consists of binding sites for phosphoinositides, rap1 GTPases, F-actin and attach to a rod comprising binding sites for integrin, vinculin, actin, KANK and others, many of which are mechanosensitive and can only be exposed by tensile forces [168]. The association of the transmembrane domain (TMD) of αIIb and β3 is maintained by specific helical packing TMD interactions near the outer membrane clasp [169], which could be disrupted by talin by altering the topology of the β3 TMD [167,170]. The direct experimental evidence suggested that talin binding to β3-integrin could change the membrane embedding and therefore the topology of integrin β3 TMD [170]. Proline-induced kink in β3-TMD could break the continuity of the helix and replace the inner membrane clasp interaction [167], which exerts crucial effects on regulating the TMD topography. Similarly, proline-induced kink can also impair talin-mediated α4β7 activation [171]. The β2 cytoplasmic tail binding to talin-1 can induce a conformational change and result in a change in the angle of the β2 TMD, which is further transmitted to the extracellular domain and leads to an extension conformation [172]. Recent studies have indicated that introducing the proline mutation L697P kink into the β2 TMD can completely affect the change in the extracellular domain of β2 conformation and prevent β2 integrin extension. Talin-mediated integrin activation is sufficient for inside-out signaling, which could be interfered with by α-actinin in a type-specific way. α-actinin plays opposite roles in controlling the activation of αIIbβ3 versus α5β1 integrin by regulating the conformation of TMD [174]. It was reported that α-actinin could impair integrin signaling by competing with talin for binding to the β3-integrin cytoplasmic tail and further inducing a kink in the TMD of β3-integrin, whereas it could promote talin binding to β1 integrin by restricting cytoplasmic tail movement and reducing the binding entropic barrier [173]. Unlike talin binding to the membrane-proximal NPXY (Asn-Pro-x-Tyr) motif of the β subunit tail, kindlin binds to the membrane distal NXXY motif and facilitates the recruitment of the integrin-linked pseudo kinase-PINCH-parvin complex, paxillin and the Arp2/3 complex to integrins [20]. Kindlins seem to be regulated by oligomerization but not conformational autoinhibition [174], while vinculin is an autoinhibited adaptor protein with multiple binding sites for other adhesion components, such as talin, IpaA, β-catenin, paxillin, PIP2 and F-actin. Activated vinculin is rapidly recruited to the actin-binding layer from a membrane-apposed integrin signaling layer and recruits additional proteins [175,176]. Paxillin is a key adaptor protein regulated by phosphorylation, which contains binding sites for adhesion, including parvin, Src, FAK, actopaxin, vinculin, talin, and ILK [177]. FAK is a

cytoplasmic tyrosine kinase that is activated by disruption of an autoinhibitory intramolecular interaction and phosphorylates substrates such as paxillin, promoting additional protein docking sites regulating downstream events [178]. The “inside-out” pathway receives priming signals from adhesion molecules, chemokine receptors and other intracellular signals. Integrin activation involves various intracellular signaling proteins described above and with other proteins, including spleen tyrosine kinase (SYK), Bruton's tyrosine kinase (BTK), phosphoinositide 3-kinase (PI3K), Rap1-interacting adaptor molecule (RIAM) and associated interacting adapter molecules, allowing subsequent downstream signal transduction [179]. For example, in neutrophils, chemokine attachment with G protein-coupled receptors (GPCRs) causes heterotrimeric G-proteins to divide into G_{α} and $G_{\beta\gamma}$, which initiates phospholipase C (PLC) activation to activate calcium and DAG signals and then promotes PI (4,5) P2 binding to activated RAP1 and RIAM via the PKC-phospholipase D (PLD)-Arf6 axis. This process induces the recruitment of talin-1 and subsequently Kindlin-3 in combination with β2 integrin [180]. Activated talin is recruited to the cell membrane and binds to induce integrin activation by stimulation with T-cell receptor (TCR) or chemokine receptors, which conduct receptor signaling to downstream cellular events such as migration and chemotaxis [181].

Outside-in signaling

Transmembrane connections and mechanotransduction. Cell invasion and migration induced by integrin-mediated adhesion complexes are involved in disease states such as tumor metastasis, autoimmune diseases and other important physiological processes [182-185]. Before adhesion formation, integrins first form tiny clusters at the junction of the cell-ECM. This is sometimes due to the transverse interaction of certain integrins across the membrane domain. These formed and dissolved clusters are regulated by the cell microenvironment [186]. Through activation of specific integrin receptors, key adaptor, cytoskeleton and kinase assemble at the cell membrane to form adhesion complexes that transduce signals from the ECM to the interior of the cell. Following integrin activation, the protein complexes consisting of integrin, adaptors, scaffolding molecules, structural proteins, protein kinases, phosphatases, and GTPases are termed IACs [186,187]. The proteomic differences between active and inactive IACs show a striking 64% similarity [188]. Active IACs have stable microtubules that participate in FA disassembly and inhibit their oligomerization. However, inactive IACs have a large number of Ras homology (Rho) and Ras GTPase family proteins, which activate myosin contractility, promoting FA maturation [189]. Further analysis identified 60 core proteins in IACs, termed the ‘consensus adhesome’, comprising four potential axes viz. FAK-paxillin, ILK-PINCH-kindlin, α-actinin-zyxin-vasodilator-stimulated phosphoprotein (VASP) and talin-vinculin [6,22,190,191]. However, Kank2-paxillin and liprin-b1-kindlin have been revealed as new associations. In parallel studies, Kank1 was localized to the periphery of mature IACs by binding talin, coordinating the formation of cortical microtubule stabilization complexes, including ELKS, liprins, kinesin family member 21A (KIF21A), LL5b and cytoplasmic linker-associated proteins (CLASPs), which in turn led to IAC instability [192,193]. Thus, Kank proteins are also considered possible core adhesome components. IACs are heterogeneous without uniform standard definition. According to size, composition, lifetime, cellular distribution and function, IACs have been classified as nascent adhesions, focal complexes, FAs, invadosomes (podosomes and invadopodia), and reticular adhesions [187]. Among them, FAs and FA-like structures are the most representative and well-studied. According to the different stages of cell adhesion to the ECM, classical FAs are preceded by focal complexes and followed by fibrillar adhesions with different molecular compositions [194-196]. “Nascent adhesions” or “focal complexes” are the earliest FA-like structures

visible under the light microscope and consist of fewer proteins, such as talin, paxillin, α-actinin and kindlin2, than typical FAs [197]. The actin polymerises in nascent adhesions cause retrograde actin flow, starting centripetal from the lamellipodium, which generates force in the opposite direction of the nascent adhesions triggering molecular events involving talin and vinculin that strengthen the integrin-cytoskeleton bonds leading to focal complex formation. This "molecular clutch" is essential for adhesion maturation and eventually cell migration and mechanotransduction [198-201]. It should be noted that although myosin II is not required for the formation of adhesions, its contractility plays an important role in the maturation of the same [200,202].

The formation and maturation of FAs require the participation of various proteins in different physiological and pathological contexts. Cooperation between αvβ3 and α5β1 integrins has been shown to play a role in FA maturation and cell spreading [203]. The binding of Talin to cell membranes has been proven to be essential for integrin activation and FA formation [204]. Talin, ILK, and the type Iγ phosphatidylinositol 4-phosphate [PI(4)P] 5-kinase (PIPKIγ) play a role in polarized FA assembly [205]. The binding of proteins such as paxillin, vinculin, VASP and zyxin to FAs depends on the orientation and locations of FAs [206]. This means that FA composition is dynamic, depending on the cellular microenvironment and that many proteins are regulated by the phosphorylation pathway [189,198,207,208]. As IACs mature, they either disassemble or undergo changes to their protein composition and signaling activity induced by force [209,210]. In addition to adhesion to ECM ligands, non-ECM ligands or counterreceptors on adjacent cells, integrins serve as transmembrane mechanical junctions that contact the cytoskeleton inside cells from those extracellular [211].

Mechanotransduction is known as the process by which cells sense mechanical stimuli and translate them into biochemical signals and is central to the processes primarily myosin motors, which exert forces on actin filaments anchored to cell–cell or cell-matrix adhesions and mechanosensors. Mechanosensing interacts with tyrosine kinases, and other signaling pathways play a key role in cancer, cardiovascular diseases and other diseases [212]. Integrin-ligand bonds and even all of the above interactions are transient in nature. Some nascent adhesions quickly disperse, while others persist and are trapped in the retrograde actin flow resulting from a combination of actin polymerization, contractile forces applied by myosin II motors and leading-edge membrane tension. Thus, integrin-mediated adhesions link the rearward-flowing actin cytoskeleton to the extracellular environment, allowing cells to exert and experience mechanical forces. This assembly is termed the molecular clutch [213,214]. The tensile stress caused by actin flow and integrin attachment to the ECM leads to conformational changes that result in exposure of cryptic binding and phosphorylation sites, which allows the recruitment and activation of additional proteins to further regulate downstream signaling pathways [215]. Talin and vinculin are two very important mechanosensitive proteins that regulate the link between integrins and actin. The application of force results in integrin clustering and initiates integrin downstream signaling through the coupling of integrins via talin and vinculin to the actin cytoskeleton. In turn, actin can pull on integrins, further promoting force generation. The N-terminal FERM domain of Talin binds directly to the NPXY motif at the proximal tail membrane of β-integrin. After subsequent attachment to F-actin, talin is stretched to cause a conformational change that exposes the first cryptic vinculin binding site in its rod R3 domain [216]. Vinculin interacts with talin and actin to unfold its closed, autoinhibited conformation [217], which permits transmission and distribution of mechanical force through the cytoskeleton. Vinculin and talin coordinate to stabilize each other's extended conformational states. Vinculin allows more force to be applied to Talin by linking it to actin, thereby exposing additional

binding sites reciprocally [216,218]. Among these interactions, the Ras-family small GTPase Rap1 and the Rap1 effector RIAM play a role in recruiting talin to the membrane and facilitating the conformational activation of talin [219]. The Talin rod, rather than vinculin unfolding induced by mechanical force, inhibited the Talin-RIAM interaction, suggesting that force may be a molecular switch regulating the interaction between vinculin-RIAM and talin [220]. Additionally, Yes-associated protein 1 (YAP)/transcriptional coactivator with PDZ-binding motif (TAZ) signaling has recently been recognized as an important mechanotransducing hub that contributes to integrating cellular and tissue mechanics with metabolic signaling, allowing transcriptional responses [221].

Integrin-mediated downstream events. As the transmembrane connection of integrins has been characterized, integrin signaling has been reported to not only modulate IACs formation and actin cytoskeletal rearrangements but also regulate intracellular pathways in response to the ECM or other ECM that triggers “outside-in” signals that serve to modulate gene expression, proliferation, survival/apoptosis, polarity, motility, shape, and differentiation [166]. Integrins engage with extracellular activators such as divalent cations, endogenous agonists, activating antibodies, and ligand-mimicking molecules [222-225], and their subsequent clustering leads to the activation of SYK, FAK and Src family kinases (SFKs), regulating integrin downstream signaling pathways [226]. In addition, mechanical forces can also trigger integrin conformational changes downstream [39,227-230]. Integrin ligation triggers the upregulation of P53 activation, BCL-2 and FLIP prosurvival molecules [231,232], and the activation of the mitogen-activated protein kinase (MAPK)/extracellular signal-regulated kinase (ERK) pathway, PI3K/AKT pathway, JNK16 signaling, and stress-activated protein kinase (SAPK) or nuclear factor κB (NF-κB) signaling [233-235]. In fibroblasts, integrin-mediated adhesion activates FAK as well as the sodium–proton antiporter and protein kinase C (PKC) [236], and recruitment of FAK to integrins has been considered to precede talin recruitment [237]. Integrin-FAK signaling is required for microtubule stabilization [238], leading to anoikis resistance in normal cells and metastasis of independent anchorage growth in tumor cells [239]. FAK interacts with a scaffolding protein, and the hematopoietic PBX-interacting protein (HPIP/PBXIP1) in FAs leads to MAPK activation, which leads to Talin proteolysis and contributes to the regulation of cancer cell migration [187,240-244]. In autosomal dominant polycystic kidney disease, increased ECM fibrosis activates the mechanistic target of rapamycin (mTOR) pathway through the ILK/PINCH/αParvin/FAK complex, further accelerating the repair of EMT and cell migration [245].

The activation of Src family kinases is one of the earliest stages of “outside-in” signaling [246]. Interaction of integrins with urokinase plasminogen activator receptor (uPAR) activates Rho GTPase to promote cell migration and invasion. α subunit of αvβ3 coupled to Fyn and Yes. Fyn and Yes activate FAK, which is a necessary element in Src homology and collagen homology (SHC) activation. SHC combined with Ras/ERK/MAPK are activated from αvβ3/receptor tyrosine kinase (RTK) receptor combinations, thus activating matrix metalloproteinases (MMPs). Neuropilins (NRPs), vascular endothelial growth factor (VEGF) receptors known as therapeutic targets of tumor growth and metastasis, promote tumorigenesis in breast cancer cells by localizing to FAs and binding to α6β1 integrin to activate FAK/Src [247]. FAs regulate turnover and cell mobility through microtubules, and autophagy and ubiquitination are equally important for their role as biosensors of the cellular microenvironment and for migration [189]. Hypoxia induces anoikis resistance by regulating activating transcription factor 4 (ATF4) and autophagy genes via the integrin signaling pathway. Cell separation from the ECM also triggers integrin signalling via the eukaryotic translation initiation factor 2 alpha kinase 3 (EIF2AK3)-reactive oxygen species (ROS)-ATF4 axis,

promoting autophagy and developing anoikis resistance [248]. RIAM-VASP relays integrin complement receptors in outside-in signaling driving particle engulfment by determining ERK phosphorylation and its kinetics [249]. In tandem with the ERK1/2 and c-Jun N-terminal kinase (JNK)1/2 pathways, β1 integrin/FAK/Cortactin pathway signals in FA disassembly and turnover, leading to cell survival and therapeutic drug resistance [250,251]. Specific mechanical cues, such as rigid environments, lack of spatial constraints and tensile loading, promote YAP/TAZ nuclear translocation and transcriptional activity [252]. Hippo-YAP signaling depends on the Enigma protein family and FAK, which signal to Hippo through the PI3K pathway [253]. Similar to the biophysical cues required for YAP/TAZ activation, myocardin-related transcription factor (MRTF) achieves transcriptional regulation of serum response factor (SRF) by translocating to the nucleus. Mechanistically, MRTFs respond to the G/F-actin ratio because G-actin binds MRTFs to promote nuclear export and sequester the protein in the cytoplasm [254]. Notably, different integrins regulate downstream signaling pathways through divergent binding mechanisms, such as latent TGF-β (L-TGF-β), a latent form of TGF-β, binding to avβ6 integrin triggers a conformational change from extended-closed to extended open, which allows actin cytoskeletal force to be transmitted through the β subunit to release mature TGF-β from its latent complex [255], while the αvβ8 has a distinct cytoplasmic domain without interacting with the actin cytoskeleton, and αvβ8-mediated TGF-β activation directs TGF-β signaling to the opposing L-TGF-β/glycoprotein A repetitions predominant (GARP)-expressing cell through the formation of a large multicomponent cell–cell protein complex [256]. A schematic overview of integrin activation-associated signaling cascades is shown in Fig. 4.

INTEGRIN ROLES IN PHYSIOLOGY AND PATHOLOGY

Integrin roles in cancer

Integrins regulate cell proliferation, adhesion, migration and survival, and tumors can hijack integrin-facilitated biological signaling to participate in every step of cancer progression, including tumor initiation and proliferation, invasiveness, circulating tumor cell survival, metastatic niche formation, immunosuppression, and colonization of the new metastatic site and support multiple therapy resistance [257]. Integrins are considered therapeutic targets in multiple cancers. The expression of integrins can vary considerably between normal and tumor tissue and is also associated with cancer types and organotrophic metastasis. For example, integrins αvβ3, αvβ6 and a5β1 are usually expressed in most normal epithelia at low or undetectable levels but can be highly upregulated in multiple tumors [258]. The overexpression of the integrins αvβ3, αvβ5, αvβ6, a5β1, a6β4, and a4β1 promotes cancer progression in various cancer types. The expression and function of major integrins and their related cancer types and metastatic sites are shown in Fig. 5, which indicates the applicability of these integrin receptors as therapeutic targets and underlines the requirement for patient stratification in future clinical studies. Herein, we summarize the recent progress in the engagements of integrins and integrin-regulated mechanisms in different cancers.

Integrin and tumorigenesis. Most integrins act as tumorigenesis promoters in multiple solid tumors, but some integrins also act as suppressors in tumor tumorigenesis [257]. The β1 integrin family has heterogeneity in tumor initiation and progression [259,260]. Several studies have suggested a beneficial role for the inhibition of β1 integrin or deletion of the β1 gene, including reversion of the malignant phenotype in breast cancer and reduction of drug resistance and metastasis in gastric, ovarian, and lung cancer [261-264]. α2β1 integrin is highly expressed on normal breast epithelium, and α2β1 integrin is reported to be a metastasis suppressor in mouse models and human breast cancer

[125]. Other studies, however, suggested integrin α2 or α2β1 as a key regulator of hepatocarcinoma cell invasion and conferring selective potential for the formation of hepatic metastasis [265]. In addition, many studies have also proven that laminin-binding integrins (α3β1 and α6β4) exert opposing effects (tumor-promoting and suppressive) on tumor development and progression [125]. Integrins may act as tumor suppressors by activating TGF-β and exerting anti-proliferative effects in the early stage of tumor formation until the cancer becomes refractory, and the inhibitory effect of TGF-β on tumor cell proliferation will decrease or even disappear; then, the same integrins can drive tumor progression [266,267]. β1 integrin expression and function are associated with metabolic reprogramming. An array of studies has suggested that glycolytic enzymes affect β1 integrin expression, which produces a vicious cycle for promoting cancer progression [268]. In colon cancer cells, the glycolytic enzyme pyruvate kinase M2 induces metabolic reprogramming, positively affecting the overexpression of enhanced β1 integrin expression and increasing cell migration and adhesion [269]. Inhibition of glycolytic enzymes could decrease integrin β1 expression and proliferation in breast cancer cells [268,269].

Integrins also play an important role in regulating immune response during tumor development [270]. Importantly, as a gut-tropic molecule, integrin α4β7 plays a profound role in regulating the progression of colorectal cancer (CRC) [271]. α4β7 mediates the recruitment of IFN-γ-producing CD4+ T cells, cytotoxic CD8+ T cells, and NK cells to the CRC tissue where they exert effective anti-tumor immune responses [271]. Higher β7 expression levels are correlated with longer patient survival, higher cytotoxic immune cell infiltration, lower somatic copy number alterations, decreased mutation frequency of APC and TP53, and better response to immunotherapy [271].

Integrins have been reported to sustain intratumoral cancer stem cell (CSC) populations depending on tumor type. Prospective identification studies suggested that integrin αvβ3, α6β1 and α6β4, which are overexpressed in CSCs, promote the sustainability of self-renewal and the expansion of CSCs for tumor initiation [272]. Actually, the α6 and β3 subunits are regarded as a signature of luminal precursor cells in mammary ductal epithelium [273], and the α6 and β4 subunits are generally applied as markers to identify bipotential progenitors in normal prostate and prostate cancer in mice [274,275]. Deletion of the signaling domain of β4, which also pairs with α6, decreases the self-renewal ability of prostate tumor progenitors [275].

Integrins play key regulatory roles in neovascularization. Endothelial cells highly express a diverse repertoire of α1β1, α2β1, αvβ3, α5β1 and αvβ5 [276,277]. In particular, αvβ3 is expressed on quiescent endothelial cells at very low levels but is markedly increased during tumor angiogenesis [278]. Therefore, integrin αvβ3 antagonists can induce endothelial cell apoptosis in neovasculature without affecting the normal vasculature, which leads to many peptide-based integrin inhibitors and antibodies developed in clinical trials for cancer treatment. Integrin αvβ3 and VEGF have a synergistic signaling connection during the activation of endothelial cells and vascularization induced by interplay between VEGF and ECM molecules [279]. The anti-integrin αvβ3 antibody BV4 inhibits the phosphorylation of VEGFR2 [279], and the VEGFR2-specific inhibitor SU1498 inhibits the complex interaction between VEGFR2 and integrin β3 [280]. FAK-Src signaling is important in both αvβ3 and VEGF-associated tumor angiogenesis [243]. The crosstalk of integrin αvβ3 and VEGFR2 could be regulated by Src. Src inhibitors not only block both the phosphorylation of integrin and VEGFR2 but also complex formation between VEGFR2 and integrin β3 [281]. The interplay of integrin αvβ3 in VEGFR signaling should be considered in anti- angiogenesis drug development.

Integrin and metastatic cascade. Metastasis causes 90% of cancer deaths [282]. The "seed-and-soil" hypothesis provides insight into organ-specific metastasis. Integrins engage in the metastatic cascade, which is dependent on tumor type, stage, metastatic site, and microenvironmental influences. For breast, prostate and lung malignancies, the most frequent metastasis site is bone. The correlative evidence suggests that the role of integrins (e.g., αvβ3, α2β1, α4β1, α5β1) mediates the interactions of tumor cells with the bone microenvironment. αvβ3 has been studied most as an important integrin for bone metastasis [283]. Integrin αvβ3 was expressed at higher levels in breast cancer patients with bone metastases than in their primary tumors [284].Tumor-specific αvβ3 participates in breast cancer spontaneous metastasis to bone by mediating chemotactic and haptotactic migration towards bone factor [285]. Functional modulation of αvβ3 is also required for prostate cancer within bone metastasis and for tumor-induced bone gain [286]. In addition, αvβ3 activation depends on the recognition of specific bone-specific matrix ligands[286]. αvβ3 could be a potential marker for bone metastasis, and treatment with αvβ3 antagonists can reduce the capacity of tumor cells to colonize bone [287].

In recent years, exosomes have been recognized as the "primers" of the metastatic niche [288]. Integrins, as the most highly expressed receptors on exosomes, are major players in mediating exosome functions and especially exert important effort in guiding exosomes to spread into the prime long-distance organs to form a premetastatic niche and further support organ-specific metastasis [289]. A comprehensive proteomic investigation suggested diverse exosome-carrying integrins derived from different types of primary tumors [290]. Most notably, lung-tropic cancer cells predominantly secreted α6β1 integrins and α6β4 integrin-positive exosomes, while liver-tropic cancer cells mainly shed exosomes with a high enrichment of αvβ5 integrin [290]. Targeting exosome uptake of integrins α6β4 and αvβ5 can reduce lung and liver metastasis, respectively [290]. In prostate cancer, αvβ6 is not detectable in the normal human prostate but is highly expressed in primary prostate cancer [291]. It was reported that αvβ6 is packaged into exosomes secreted by prostate cancer cells and transferred into αvβ6-negative recipient cells, which contributes to enhancing cell migration and metastasis in a paracrine fashion [291]. αvβ3-expressing exosomes are highly enriched in the plasma of prostate cancer patients; in addition, the levels of αvβ3 remain unaltered in exosomes isolated from blood from prostate cancer patients treated with enzalutamide [292]. Exosome-carrying integrin αvβ3 is transferred to nontumorigenic recipient cells and promotes a migratory phenotype [293]. Exosome-carrying integrin α3 (ITGA3) and ITGB1 from urine from prostate cancer with metastasis are more abundant than those from benign prostate hyperplasia or primary prostate cancer [294]. In pancreatic cancer, numerous lines of evidence suggest that exosomal integrins also play key roles in exosome-mediated tumor progression and metastasis; for example, exosome-carrying αvβ5 released by primary tumor cells in the pancreas tends to metastasize to the liver, whereas α6β4 and α6β1 tend to metastasize to the lung [295]. In future studies, the general applicability of exosome integrin-mediated organ-specific metastasis remains to be validated *in vivo* models and in other cancer types.

Integrin and drug resistance. Tumor metastasis and therapeutic resistance together determine a fatal outcome of cancer. Interactions between cell surface integrins and ECM components have been found to be responsible for intrinsic and acquired therapy resistance, which is named cell adhesion-mediated drug resistance (CAMDR) [282,288]. Generally, integrins are involved in resistance to most first-line therapies in the clinic, such as radiotherapy [289], chemotherapy [290], angiogenesis [291], endocrine therapy [292], and immunotherapy [293]. The mechanism of integrin-induced primary and

adaptive drug resistance is variegated. In various cancers, β1 integrin-interacting matrix molecules promote primary radiotherapy resistance by activating DNA repair and prosurvival signaling through the engagement of FAK, SRC, PI3K-AKT and MAPK signaling [294-297]. In addition, integrin-mediated reprogramming also induces radiosensitization [289]. The interaction of Integrin with ECM by activating ATP binding cassette (ABC) efflux transporters enhances the intracellular drug concentration and promotes chemoresistance to doxorubicin and mitoxantrone [298]. Cluster of differentiation-44 (CD44), alone or together with MET receptor, also participates in the upregulation of P-glycoprotein (P-gp) expression and promotes chemoresistance [299]. In xenograft models and patient specimens, Arman et al. found that c-Met replaced α5 integrin from β1 integrin and formed the c-Met/β1 complex during metastases and invasive resistance, and decoupling the crosstalk in the c-Met/β1 complex may have therapeutic implications for antiangiogenic drug resistance [300]. The interaction of integrin αvβ3 with osteopontin engages in acquired epidermal growth factor receptor tyrosine kinase inhibitor (EGFR-TKI) resistance by activating the downstream FAK/AKT and ERK signaling pathways in EGFR mutant non-small cell lung cancer [301]. Integrins are involved in invasion, angiogenesis, bone metastases and anti-androgen resistance in prostate cancer [292]. The mechanism of resistance to androgen ablation is not well understood. In our previous study, we found that the integrin-ECM interaction promotes enzalutamide (anti-androgen drug) resistance in castration-resistant prostate cancer (CRPC) via the PI3K/AKT and ERK1/2 pathways [302]. αvβ3 and αvβ6 expression are required for prostate cancer progression, including CRPC. Integrin αvβ6 can induce androgen receptor (AR)-increased activity in the absence of androgen via activation of JNK1 and further upregulation of survival [303]. In mouse melanoma and breast cancer models, Tregs expressing integrin β8 (ITGB8) are the main cell type in the tumor microenvironment, which activates TGF-β produced by cancer cells and promotes immune escape, and ITGB8 ablation or anti-ITGB8 antibody treatment could improve cytotoxic T-cell activation [293]. In triple-negative breast cancer (TNBC), integrin αvβ6 on the surface of tumor cells activates TGF-β, and upregulating SRY-related HMG box (SOX) 4 transcription factor contributes to immunotherapy resistance. An integrin αvβ6/8-blocking monoclonal antibody can inhibit SOX4 expression and sensitize TNBC cells to programmed cell death ligand 1 (PD-1) blockade [304]. Therefore, targeting integrin is regarded as a promising therapeutic opportunity for overcoming multiple drug resistance.

Integrin roles in fibrotic diseases

Fibrosis refers to chronic inflammation or injury induced by various factors, resulting in an increase in fibrous connective tissue and a decrease in parenchymal cells. It causes abnormal structural changes and functional abnormalities in injured organs, which is an abnormal manifestation of excessive damage repair [305]. Fibrosis occurs in almost any organ, especially the liver, lung, and kidney. Fibrosis diseases are difficult to detect in the early stages, and most are found to have progressed to organ sclerosis, which can be life-threatening for patients [305]. Currently, therapies for fibrosis disease are still limited, and organ transplantation is the only effective treatment option for end-stage fibrosis diseases [306]. However, due to the limited number of donor organs and their high price, replacement therapy has not been widely used. It is particularly important to develop new antifibrotic drugs from the pathogenesis of fibrosis.

TGF-β1 plays a critical role in the pathogenesis of fibrosis and has been considered a therapeutic target for fibrotic diseases [307-309]. Unfortunately, both preclinical and clinical trials have shown that direct targeting of TGF-β1 for fibrosis disease treatment is not feasible [308]. TGF-β1 is involved in the regulation of the immune system and plays important anticancer and cardiac function

maintenance roles [308,310,311]. Global inhibition of TGF-β1 leads to serious multiple organ dysfunction [308].

Encouragingly, researchers have found that blocking the interaction between integrins (especially integrins rich in αv subunits) and TGF-β1 showed an efficient anti-fibrosis effect without causing TGF-β1 dysfunction-induced adverse effects [305]. Integrins are receptors by which cells adhere to the ECM [312]. Several integrins have been confirmed as activators of TGF-β1 [312], and antagonists of αvβ1 [54] and αvβ6 [313,314] have shown considerable inhibitory effects in experimental animal models of liver, lung, and renal fibrosis. In fact, in recent years, several integrin inhibitors have been developed and evaluated in phase II and III clinical trials in fibrotic diseases, such as PLN-74809, IDL-2965, GSK3008348, and STX-100 [315]. These findings revealed the promise of integrin inhibitors in the treatment of fibrotic diseases. In the following, we focus on nonalcoholic steatohepatitis (NASH), pulmonary hypertension (PH), and autosomal dominant polycystic kidney disease (ADPKD), the diseases that usually cause fibrosis, and discuss the role of integrins in fibrotic processes (Fig. 6).

NASH. NASH, a chronic liver disease that develops from nonalcoholic fatty liver disease (NAFLD), is one of the most common chronic liver diseases in patients without a history of alcohol abuse [316,317]. Approximately 30~40% of NASH patients develop fibrosis, and 10% develop cirrhosis [318]. The prognosis of NASH depends on histological severity, especially hepatic fibrosis [319]. Therefore, preventing the progression of NASH to liver fibrosis is of great importance in NASH treatment. Despite the increasing incidence of NASH-related liver fibrosis, which currently kills 2 million people worldwide each year [320-322], there are no approved drugs. Most drugs in clinical trials target the early stages of steatosis/hepatitis other than fibrosis itself, which generally result in inadequate outcomes [323,324]. This dilemma provides an opportunity for integrin inhibitors to be applied in the treatment of liver fibrosis [28]. Several integrins have been identified to inhibit the progression of NASH to liver fibrosis, including αvβ3, α4β7, α9β1, and α8β1 (Fig. 6).

Integrin αvβ3 is expressed in hepatic stellate cells (HSCs) [325], which are considered key mediators of fibrotic responses [326]. Generally, integrin αvβ3 induces myofibroblast cells to express α-smooth muscle actin (α-SMA), leading to excessive production of ECM [327,328]. It has been reported that integrinαvβ3 and αvβ5 bind with secreted osteopontin in the liver of NAFLD mice, which inhibits autophagosome-lysosome fusion and promotes lipid accumulation [329]. Application of osteopontin antibody not only suppressed hepatic steatosis but also attenuated liver fibrosis [329], indicating a functional role of integrin αvβ3 and αvβ5 in inhibiting the progression of NASH to liver fibrosis. Moreover, in high glucose-induced human liver sinusoidal endothelial cells (HLSECs) (an *in vitro* model of NAFLD), integrin αvβ3 antibody (clone LM609) significantly downregulated the expression of laminin and suppressed fibrosis [330]. In fact, numerous studies have confirmed the efficacy of integrin αvβ3 as a predictor of fibrosis in experimental NASH models [325,328,331]. However, no integrin αvβ3 inhibitors have been evaluated in clinical trials to investigate their inhibitory effect on the progression of NASH to liver fibrosis. It is waiting to be explored.

Integrin β7 expressed in leukocytes is regarded as an important receptor that binds to MAdCAM-1 and induces homing of leukocytes to gut-associated lymphoid tissue [332]. Integrin β7 pairs with other integrin α subunits, including α4 and αE [332], in which α4β7 affects the progression of NASH to liver fibrosis [332-334]. At first, researchers focused only on the role of integrin β7 in NASH-induced liver fibrosis. Knockout of integrin β7 (ITGB7*)* significantly promoted inflammatory cell infiltration and fibrosis in the livers of NASH mice [332]. In contrast, MAdCAM-1

knockout showed anti-inflammatory activity [332]. Later, integrin α4β7 was found to play an important role in the progression of NASH to liver fibrosis. The abnormality of gut microbiota in NASH mouse models promoted the expression of MAdCAM-1 in the liver, which recruited α4β7-positive CD4 T cells to the liver and induced inflammation and fibrosis [334]. Blocking integrin α4β7 has shown promising therapeutic effects on fibrosis in NASH [334], indicating its great potential as a therapeutic target for NASH-induced liver fibrosis.

Integrin α9β1 plays an important role in lipotoxic hepatocyte-induced hepatic recruitment of monocyte-derived macrophages (MoMFs), which promotes the progression of NASH to fibrosis [335]. Integrin α9β1 expressed in hepatocytes could be activated by hepatocyte lipotoxicity and endocytosed by hepatocytes [335]. Extracellular vesicles are formed and secreted by hepatocytes, which are further captured by MoMFs [335]. Integrin α9β1 mediates MoMF adhesion to liver sinusoidal endothelial cells by binding to VCAM-1, which induces inflammation [335]. Blocking integrin α9β1 significantly reduced liver injury, liver inflammation, and liver fibrosis [335], indicating that it is a therapeutic target for fibrosis in NASH. In addition, it has also been reported that anti-mouse osteopontin mouse IgG (35B6) inhibits the cell adhesion of mouse and human osteopontin to Chinese hamster ovary (CHO) cells expressing integrin α9, which suppresses liver inflammation and fibrosis in NASH mice [336]. All these findings revealed the therapeutic potential of integrin α9β1 inhibitors in liver fibrosis induced by NASH.

Integrin α8β1 is expressed in smooth muscle cells, HSCs, and fibroblasts [337]. It was upregulated in patients with NAFLD and liver fibrosis [82,338]. In NASH, the activation of HSCs expressing the integrin α8 subunit has been proven to be an agonist of latent TGF-β, which participates in promoting fibrosis [82]. A previous study showed that inhibiting the integrin α8 subunit with an integrin α8 antibody significantly improved liver fibrosis in a NASH mouse model [82]. In addition, miR-125b-5p silencing caused by NAFLD also downregulated integrin α8, which inhibited the RhoA signaling pathway and promoted fibrosis [338]. These results implied the functional role of integrin α8β1 in promoting liver fibrosis induced by NASH.

Moreover, other integrins have also been proven to be involved in liver fibrosis. Integrins containing the αv subunit have received the most attention due to their activating activity on TGF-β, including αvβ1, αvβ5, αvβ6, and αvβ8 [306,327]. In addition, integrins α11 and RGD-recognizing integrins (such as αIIbβ3 and α5β1) are also important regulators of liver fibrosis [339]. Integrin inhibitors such as IDL-2965 and PLN-74809 have been investigated in clinical trials to evaluate their therapeutic effect on liver fibrosis [339]. However, none of their roles in fibrosis induced by NASH have been elucidated. It may be a promising direction for the treatment of NASH-derived liver fibrosis.

PH. PH is a disorder of the pulmonary vasculature defined by increased pulmonary vascular resistance ≥3 Wood units [340]. It is characterized by excessive pulmonary vasoconstriction and vascular remodelling resulting in persistent elevation of pulmonary arterial pressure [341]. PH causes right ventricular hypertrophy, right heart dysfunction, and even right heart failure, threatening up to 100 million people worldwide [340,342]. Pulmonary vascular remodelling in PH involves the processes of endothelial injury, endothelial cell abnormality, excessive vascular smooth muscle cell proliferation, invasion of the intima by (myo)fibroblast-like cells and, especially, intimal fibrosis [343]. Increased deposition of interstitial ECM components, including collagen, elastin, tenonin-C, and fibronectin, has been demonstrated in human patients and animal models [341,344-346]. As the receptor for ECM proteins, integrins play important roles in maintaining vascular remodelling [347].

Pulmonary vasculature expresses several types of integrins, including α1, α2, α3, α4, α5, α7, α8, αv, β1, β3, and β4 [12,348,349](Fig. 6). Studies revealed that in the pulmonary arteries (PAs) of chronic hypoxia and monocrotaline-treated PH rat models, integrin α1, α8, and αv were upregulated, and integrin α5, β1, and β3 were downregulated significantly [347,350]. Integrin αv activates TGF-β1 and TGF-β3, which are critical to vascular homeostasis. TGF-β regulates PH through multiple signalling pathways, including upregulation of endothelial nitric oxide synthase, stimulation of VEGF and endothelin-1, alteration of bone morphogenetic protein (BMP) signalling, and anaplastic lymphoma kinase (ALK)-1–ALK-5 signalling in endothelial cells [351-353]. Integrins β1 and β3 have been reported to regulate cell proliferation by interacting with activated ILK, a pro-proliferative protein kinase. ILK is activated by integrins in response to growth factors and cytokines, which in turn trigger downstream signals, including activation of Akt and inhibition of the growth suppressor HIPPO [354-356]. ILK1 is upregulated in pulmonary artery vascular smooth muscle cells (PAVSMCs) of human pulmonary arterial hypertension (PAH) and experimental models and is required for increased cell proliferation, survival, pulmonary vascular remodelling, and overall PH, and inhibition of ILK reverses experimental PH in male mice[355]. Researchers believe that integrin α1 and α5 may participate in regulating ECM, as they are expressed in the smooth muscle cells of PAs (PASMCs) [347]. In these processes, integrin α1-ligand collagen IV expands, while integrin α5-ligand fibronectin suppresses chronic hypoxia treatment-induced FAK phosphorylation [347]. The regulatory effects of integrin α1 and α5 on FAK phosphorylation then react to Ca^{2+} signaling, which may be involved in intimal fibrosis [347].

In addition, integrin β3 may function as an inhibitor of fibrosis and vascular remodelling in PH. It has been reported that silencing integrin β3 (ITGB3) significantly improves chronic hypoxia-induced pulmonary hemorrhage, pulmonary vascular remodelling, and pulmonary fibrosis in rats [350]. These effects may come from the interaction between integrin β3 and ECM. However, the underlying mechanism still needs to be clarified. The role of integrin αv in regulating PH-induced fibrosis has attracted little attention. However, the interaction between αvβ3 and osteopontin has been confirmed, which activates FAK and AKT, promoting the proliferation of PASMCs and enhancing vascular remodelling [357,358].

ADPKD. ADPKD is an autosomal dominant kidney disease caused by polycystic kidney disease-1 (PKD1) or polycystic kidney disease-2 (PKD2) gene mutations. It is the fourth leading cause of end-stage renal disease (ESRD), with an incidence of approximately 1/2500 to 1/1000 [359,360]. ADPKD is characterized by progressive growth of multiple renal tubules and collecting duct-derived cysts in bilateral kidneys, which compress the renal parenchyma and cause nephron loss [361]. Fibrosis is an important pathophysiological change of ADPKD that directly leads to renal dysfunction and induces ESRD [359]. Therefore, antifibrosis is important in the treatment of ADPKD. However, apart from replacement therapies, there is no clinical solution that could effectively prolong the lifespan of ADPKD patients, which makes it urgent to develop new drugs [362].

In recent decades, research on integrin function in fibrotic kidney diseases has achieved exciting results. A growing number of integrins have been found to play regulatory roles in the progression of fibrosis in renal dysfunction and show great potential as therapeutic targets for renal disease. In particular, integrin αvβ3 [245] and β1 [363] are promising antifibrotic targets in ADPKD treatment (Fig. 6).

As an important activator of latent TGF-β1, integrin αvβ3 enhances TGF-β/small mothers against decapentaplegic (SMAD) signaling pathways, which induces ECM production, promoting

renal fibrosis in ADPKD [245]. Periostin is a ligand of integrin αvβ3, which binds to integrin αvβ3 through its fasciclin 1 (FAS1) domains and promotes the release of TGF-β from latent TGF-β-binding protein [245]. Periostin (Postn) has been confirmed as a profibrotic factor and was upregulated in ADPKD [364]. Studies reported that global knockout of postn in pcy/pcy mice, an ADPKD mouse model, significantly inhibited renal cyst development and renal fibrosis [365]. In contrast, overexpression of periostin obtained the opposite results [366]. All these effects of periostin on fibrosis in ADPKD were thought to be mediated by integrin αvβ3 [364-366]. Recently, osteopontin was reported as a urinary biomarker for predicting ADPKD progression [367]. Since osteopontin is another ligand that activates the interaction between integrin αvβ3 and TGF-β1, this study seems to confirm the profibrotic effects of integrin αvβ3 in ADPKD.

Integrin β1 is the most prevalent β-chain integrin subunit expressed in the kidney [368]. It has been reported that knockout of ITGB1 significantly ameliorates renal fibrosis by suppressing the expression of α-smooth muscle actin (α-SMA), fibronectin, and collagen in the kidneys of PKD1 knockout mice [363]. Several integrins that contain the β1 subunit have been identified as regulators of renal fibrosis, including α1β1 [369], α2β1 [370], α5β1 [371], and αvβ1 [372]. Although whether these integrins function in the fibrotic process of ADPKD has not been fully elucidated, their great potential to be developed as an antifibrotic target for ADPKD treatment could not be neglected.

In addition, integrins contain αv subunits (such as αvβ5 [373] and αvβ6 [374]), and integrin α3 [375] also participates in promoting renal fibrosis. However, the roles they play in ADPKD are unclear. However, there is no integrin inhibitor that undergoes a clinical trial to evaluate its therapeutic effects on renal fibrosis. In future studies, the profibrotic mechanism of integrins in ADPKD and evaluating their therapeutic effect on ADPKD are expected to disperse the dimness brought by ADPKD.

Integrin roles in cardiovascular diseases

Atherosclerosis. Atherosclerosis (AS) is the fundamental pathological process of vascular diseases. The rupture of atherosclerotic plaques and secondary thrombosis are the most common causes of severe vascular events. The alteration of integrin signaling pathways can affect multiple aspects of AS, such as endothelial dysfunction and activation, leukocyte homing to the plaque, leukocyte function within the plaque, smooth muscle recruitment and fibroproliferative remodelling, and thrombosis [376]. In view of the crucial role of integrins in the occurrence and development of AS, we review the integrin regulation of AS and the potential of integrins as therapeutic targets. The model for atherosclerotic plaque development and the main roles of integrins in the process of AS are shown in Fig. 7.

Oxidized low-density lipoproteins (Ox-LDL) and shear stress generated by blood flow lead to endothelial cell dysfunction, which in turn promotes inflammatory cell homing and infiltration. Monocytes migrate into the subendothelium, transform into macrophages and initiate AS. Ox-LDL can activate α5β1 and induce α5β1-dependent signal transduction, thereby activating the FAK/ERK/p90 ribosomal S6-kinase (p90RSK) pathway to induce NF-κB signaling [37[illegible]]. Shear stress activates provisional matrix binding integrins (α5β1 and αvβ3), and some studies have reported that αvβ3 inhibition is sufficient to prevent NF-κB activation involving p21-activated kinase (PAK) signaling on fibronectin [378,379]. In addition, proinflammatory gene expression (ICAM-1 and VCAM-1) also increases after ox-LDL and shear stress-induced ligation of provisional matrix-binding integrins [377,380].

Leukocytes express integrins that mediate interactions with cell adhesion molecules on endothelial cells. Several studies have shown that α4β1 and various β2 integrins play vital roles in the formation of atherosclerotic plaques. α4β1 is the major leukocyte VCAM1 receptor [381]. αxβ2 and α4β1 can bind VCAM-1 cooperatively to promote leukocyte adhesion [382]. In addition, αxβ2 and αLβ2 interact with ICAM1/2 on the surface of endothelial cells. Deficiency of αx integrin significantly reduces monocyte recruitment and AS development in apoE-/- hypercholesterolemic mice [383]. Monocyte integrins α4β1, α9β1, and αvβ3 interact with osteopontin, which is expressed in atherosclerotic plaques, to promote monocyte migration and survival [384]. Integrin αDβ2 shows prominent upregulation during macrophage foam cell formation [385]. Meanwhile, ligation of specific macrophage integrins (e.g., αMβ2, αvβ3) may affect various aspects of macrophage function in AS [376], including macrophage clearance of local lipid deposits [386-388], phagocytosis of apoptotic cell debris [389,390] and the ability to promote local proinflammatory gene expression [391]. Recently, nexinhib20, a neutrophil exocytosis inhibitor, has been confirmed to inhibit exocytosis and neutrophil adhesion by limiting β2 activation [392], which sheds new light on targeting integrin β2 therapy.

Vascular smooth muscle cells (VSMCs) are vital in the progression of AS because they can transdifferentiate into proliferative and migratory phenotypes. Current studies support the key role of αvβ3 signaling in smooth muscle proliferation and migration. Both α5β1 and αvβ3 bind to fibronectin, and their inhibitors reduce atherosclerotic plaque formation, but only αvβ3 inhibition reduces fibrous cap formation incidence [378,393]. Ligation of αvβ3 and αvβ5 integrins mediates FAK activity [394] and causes VSMC migration by AKT and paxillin phosphorylation [395-397].

The rupture of an atherosclerotic plaque is the primary trigger for arterial thrombosis. Platelets express integrins of the β1 and β3 families (α2β1, α5β1, α6β1, αvβ3, and αIIbβ3), whose main ligands are collagen, fibronectin, laminins, vitronectin, and fibrinogen, respectively [398]. Platelet adhesion promoted by α2β1 induces αIIbβ3 activation by the phospholipase C-dependent stimulation of the small GTPase Rap1b [399]. Inactive aIIbβ3 on resting platelets is conformationally converted into active to bind fibrinogen, triggering platelet aggregation and augmenting thrombus growth.

Although integrin signaling has been found to be involved in multiple developmental stages of AS, there are still a wide range of pathological processes that need to be further explored. Future studies should focus on more selective integrin inhibitors and explore better ways to target integrin inhibitors to specific cell types to establish the worth of integrins as therapeutic targets for reducing AS and its complications.

Thrombosis. Thrombosis can occur in the arterial or venous circulation and has become a major health issue associated with high morbidity and mortality [400]. Arterial thrombosis caused by rupture of atherosclerotic plaque has been mentioned above.

αIIbβ3 is the most abundant integrin in blood platelets [401] and is critical for arterial thrombosis [402]. It binds to fibrinogen by the HHLGGAKQAGV sequence in the C-terminus of the fibrinogen γ chain and RGD sequences in the α chain [398]. Inside-out signaling activates αIIbβ3, which contributes to platelet adhesion and aggregation. Outside-in signaling mediates platelet spreading and amplifies platelet thrombi [403-406]. Therefore, αIIbβ3 antagonists, which are designed to block the ligand binding function of αIIbβ3, are able to treat thrombosis, such as three current FDA-approved antiplatelet agents (abciximab, eptifibatide and tirofiban). Numerous oral compounds (orbofiban, sibrafiban, xemilofiban, lefradafiban, and roxifiban) have undergone substantial research. Because

of adverse effects such as increasing cardiovascular events, oral active antagonists have not yet received approval [24].

Compared to αIIbβ3, αvβ3 is widely expressed in tissues in addition to platelets [407]. A growing number of studies have shown that integrin αvβ3 is essential for mediating the adhesion of monocytes, platelets and endothelial cells. One of the key regulators of pathological angiogenesis and endothelial function is generally αvβ3 integrin [408-410]. In vivo, it is expressed at low levels on quiescent endothelial cells but is markedly increased during wound angiogenesis, inflammation, and tumor angiogenesis [279]. In vitro, αvβ3 mediates the adherence of platelets to osteopontin and vitronectin [411]. It is also involved in the regulation of endothelial cell function [412,413], platelet aggregation and thrombosis [414,415]. Moreover, clinical studies suggest that genetic variants of integrin β3 may be used to predict venous thromboembolism in colorectal cancer patients [416]. Therefore, integrin αvβ3 is an emerging approach for the identification and treatment of thrombotic-related diseases. Further research is still required to determine its reliability and specific mechanism.

In addition to integrins expressed on platelets, α9β1, which is highly expressed in neutrophils, is also involved in thrombosis via several mechanisms [417-419]. α9β1 is upregulated during neutrophil activation and interacts with VCAM-1 and polymeric osteopontin to mediate neutrophil chemotactic activity and stabilize adhesion to endothelial cells, leading to an increased risk of thrombosis [420,421]. Moreover, apoptosis of neutrophils is inhibited by α9β1 through the PI3K and ERK signaling pathways [422]. Integrin α9 can also modulate arterial thrombosis by enhancing NETosis. Treatment with anti-integrin α9 antibody in wild-type mice inhibits arterial thrombosis, thereby revealing a novel role for integrin α9 in the modulation of arterial thrombosis [423]. Due to the importance of both neutrophils and neutrophil extracellular traps for deep vein thrombosis and chronic thrombosis [424-426], it may be a promising line of research to explore the role of α9β1 in venous thrombosis.

Cardiac hypertrophy. Cardiac hypertrophy is defined as an increase in the size of cardiomyocytes. It is initially an adaptive response to physiological and pathological stimuli, but pathological hypertrophy usually progresses to heart failure [427]. Hypertrophy is directly related to β1 integrin, including β1A and β1D [428,429]. Deficiency of integrin β1 induces hypertrophic changes with reduced basal contractility and relaxation [430] and increases myocardial dysfunction after myocardial infarction [431]. A previous study showed a correlation between the expression of integrin β1 and angiotensin II type 1 (AT_1) receptor. An AT_1 blocker could promote the regression of cardiac hypertrophy by reducing integrin β1 expression [432]. Moreover, a β3 integrin/ubiquitination (Ub)/NF-κB pathway has been identified to contribute to compensatory hypertrophic growth [433]. FAK plays a key role in further proceeding the intracellular signals after integrin activation [434,435]. Moreover, melusin, a muscle-specific integrin β1-interacting protein, is important in protecting cardiac hypertrophy [436,437]. ILK also emerges as a crucial player in mechanotransduction by integrins [438,439].

Cardiac hypertrophy is not autonomous and is entirely dependent on events occurring in muscle cells. Macrophages can also potentially contribute to the pathogenesis of cardiac hypertrophy. Integrin β2 contributes to the adhesion of macrophages to endothelial cells, and β2 blockade attenuates cardiac hypertrophy in mice [440]. The mechanism of integrins in cardiac hypertrophy needs to be further understood and explored, such as differences in signaling pathways that initiate compensatory and decompensated cardiac hypertrophy. Targeting integrins and signaling pathways may be novel strategies to control cardiac hypertrophy and prevent heart failure.

Integrins play vital roles in myocardial fibrosis. The expression and function of integrins are altered in the diseased heart [441]. Targeting integrins and their associated proteins can be a potential

therapeutic target for myocardial fibrosis. Scar tissue size following heart injury is an independent predictor of cardiovascular outcomes [442]. The differential expression of integrins αvβ3 and αvβ5 in cardiac fibroblasts of collagen V-deficient mice drives myofibroblast differentiation, and a specific inhibitor, cilengitide, can rescue the phenotype of increased postinjury scarring [443]. Integrins are also involved in aneurysms. The expression of both α5 and αv subunits in VSMCs plays an important role in assembling ECM within the vessel wall, and the loss of these two integrins leads to the formation of large aneurysms within the brachiocephalic/carotid arteries [444]. Thoracic aortic dissection (TAD) is also associated with integrins. Macrophage-derived legumain binds to integrin αvβ3 in VSMCs and blocks it, thus attenuating Rho GTPase activation, downregulating VSMC differentiation markers and ultimately exacerbating the development of TAD [445].

Integrin roles in infectious diseases

SARS-CoV-2 infection. Severe acute respiratory syndrome coronavirus 2 (SARS-CoV-2) is a dimeric virus in the *Betacoronavirus* genus [446]. The viral genome consists of four structural proteins, namely, spike (S), envelope (E), membrane (M), and nucleocapsid (N). The envelope, membrane and nucleocapsid are integrated into the viral envelope. A growing number of studies have focused on the integrin-mediated regulation involved in virus entry and spread (Table 1). αvβ6 integrin has been reported to be of interest in inhibiting SARS-CoV-2 entry and treating coronavirus disease 2019 (COVID-19)-related diseases [447]. SARS-CoV-2 acts on human cells through angiotensin converting enzyme II (ACE2), and recent studies suggested that integrins might be the cell receptors for SARS-CoV-2 [448]. The association between the S protein of SARS-CoV-2 and the ACE2 receptor has been established, but the S1 subunit contains a solvent-exposed RGD binding motif. It is recognized by integrins, particularly α5β1 and αVβ3 [449,450]. Moreover, the SARS-CoV-2 S protein was reported to interact with integrins independent of the RGD sequence, which helps to explain how SARS-CoV-2 and other viruses evolved to interact with integrins [451]. Viruses bind cell-surface integrins via RGD. *In vitro* studies have provided evidence of cognate binding interactions between SARS-CoV-2 S proteins, integrin β1 [452,453] and integrin β3 [454,455]. Some drugs or methods that target integrins have been shown to have effects on infection. One study suggested that the ATN-161 molecule inhibited the S protein interaction with α5β1 integrin, and the interaction of α5β1 integrin and ACE2 represents a promising approach to treat COVID-19 [453]. Mn^{2+} accelerates the cell entry of SARS-CoV-2 by inducing integrin extension and binding to high-affinity ligands [456]. In addition, integrins found on the surfaces of pneumocytes, endothelial cells and platelets may be vulnerable to SARS-CoV-2 virion binding. Below, we summarize six known integrins and their potential roles in SARS-CoV-2.

Although several approaches to integrin delivery to SARS-CoV-2 host cells have been discussed in the current literature, data from peer-reviewed experiments on this topic are still scarce. More data on integrin involvement and integrin ligands in SARS-CoV-2 infection, disease progression, and recovery are needed before clinically relevant imaging or therapeutic approaches can be realized.

Human immunodeficiency virus (HIV). Monocytes/macrophages play an important role in HIV transmission in all stages of HIV infection and disease. Adhesion molecules, including integrins, are recognized as the main factors that influence HIV viral replication. Previous studies proved that blocking αv and integrin binding triggered a signal transduction pathway, which inhibited the transcription of NF-κB-dependent HIV-1 [457]. Inhibition of β integrins (specific monoclonal antibody, small RGD mimetic compounds and RNA interference) proved that integrin β5 mainly contributed

to the blockade of HIV-1 replication[458]. Other integrins, such as αvβ3 and α4β7, have also been proven to be associated with HIV. For example, the transactivating factor of HIV-1 binds to integrin αvβ3, prompting neovascularization [459]. α4β7, as a structurally dynamic receptor, mediates outside-in signaling to cells. The HIV envelope protein GP120 binds to and signals by α4β7 [460]; thus, targeting α4β7 might be a new therapeutic method to prevent and treat HIV infection [461].

Other infectious diseases, such as the West Nile virus, enter cell entry by using the integrins αvβ1 and αvβ3 [462,463]. Ebola is related to integrin α5β1, and herpes simplex virus type 1 (HSV-1) interacts with αvβ3 [464,465]. Moreover, in immunized mice, the increased frequency of circulating integrin α4β7$^+$ cells is correlated with protection against Helicobacter pylori infection [466]. β2 integrin is important in the recruitment of dendritic cells to the infection site and may affect the initiation of innate immunity [467]. The overexpression and suppression of integrin α6 increases and decreases stemness phenotypes of HPV^{+ve} head-neck squamous cell carcinoma (HNSCC) cells, respectively [468]. Severe anti-programmed death-1 (PD1)-related meningoencephalomyelitis can be treated with anti-integrin α4 therapy [469]. Studies of murine and human cells expressing RGD-binding integrins proved that αvβ6 and αvβ8 heterodimers were involved in M1 and M3 infections [470]. These targets are of great significance for the mechanistic exploration and treatment of HIT and other infectious diseases, and more research data are needed in the future.

Integrin roles in autoimmune diseases

Integrins participate in the immune response against autoimmune diseases such as inflammatory bowel disease, multiple sclerosis, rheumatoid arthritis, systemic lupus erythematosus, and psoriasis, which induces strong adhesion between lymphocytes, endothelial cells and epithelial cells by binding to ECMs and specific receptors. Many integrins are expressed in T cells, B cells, neutrophils, natural killer (NK) cells, monocytes, dendritic cells, macrophages and platelets [471].

Inflammatory bowel disease (IBD). IBD comprises a series of chronic recurrent intestinal diseases, including ulcerative colitis (UC) and Crohn's disease (CD) [472]. The pathogenesis of IBD has not yet been clearly elucidated, and genetic predisposition, dysregulation of gut microbiota, or environmental factors cause an inappropriate and persistent immune response triggering impaired intestinal barrier function and stenosis [473-476]. Evidence suggests that IBD and its associated complications are not only modulated by sustained inflammation but also maintained by inflammation-independent mechanisms [477]. Integrins have been considered to be involved in both inflammatory and inflammation-independent mechanisms due to their important roles in immune cell recruitment and cell-ECM interactions in intestinal diseases [478,479].

Integrins α4β7, α4β1 and αEβ7 are mainly involved in mediating lymphocyte homing to the intestinal mucosa. Integrin α4β7 is specifically expressed on lymphocytes in the gastrointestinal tract and mediates the motility and adhesion of lymphocytes when inactive and activated, respectively [480,481 482,483]. Integrin α4β7 highly expressed on CD4$^+$ memory T cells interacts with MAdCAM-1 expressed in intestinal inflammatory foci and regulates the homing of activated T cells during inflammation [484-486]. In addition, α4β7 expression promotes the infiltration of regulatory T cells into the gut, whereas blockade reduces enteric homing of regulatory and effector T cells [480]. α4β1 integrins (found on most leukocytes) are highly expressed in lymphoid tissues of the gut and interact with VCAM-1 expressed on the endothelium [487-489]. Adoptive transfer of α4 null T cells inducing defective homing of T cells to the inflamed tissues in immunodeficient mice significantly alleviated chronic colitis [490]. Blocking α4-integrin prevents immune infiltration of the activated T-cell populations driving IBD [488,491]. Integrin αEβ7 is mainly expressed on the surface of CD8$^+$ T

cells, Treg cells, $CD69^+\alpha E^+$ intestinal tissue-resident memory T (TRM) cells, TH9 cells, and mucosal DC subsets, allowing them to adhere to the layer of the intestinal epithelium as a result of interacting with its ligand E-cadherin[492-498]. $CD8^+$ T cells remain within the intestinal epithelium by downregulating α4β7 and upregulating αEβ7 to bind E-cadherin [499,500]. Proinflammatory $CD4^+$ T cells displaying Th17 and Th1 inflammatory phenotypes highly express αEβ7 in the colon and reduce the expression of associated genes, including inducible costimulator (ICOS), cytotoxic T-lymphocyte antigen (CTL-4), interleukin-10 (IL-10), and forkhead box protein P3 (FOXP3) [489]. A subset of $CD4^+$ T cells with the natural killer group 2D (NKG2D) receptor also express integrin αEβ7, which is characterized by inflammatory and cytotoxic effects [501]. Th9 $CD4^+$ and $CD8^+$ cells expressed increased αEβ7 compared with α4β7 expressed by Th17 and Th2 T cells [496]. In the colon of UC patients, the ability of αE^+ dendritic cells (DCs) to generate regulatory T cells is attenuated and induces a Th1/Th2/Th17 phenotype in $CD4^+$ effector T cells [502]. The frequency and tolerogenic functionality of αE^+ DCs are altered in the inflamed intestinal mucosa [503]. In addition to being physically retained in the intestinal epithelium, T lymphocytes expressing αEβ7 have direct cytotoxic activity against epithelial cells [489,504], and αE expression on a subset of resident memory $CD4^+CD69^+$ T cells accumulated in the mucosa of IBD patients predicts the development of flares[495]. Blockade of β7 integrin inhibits lymphocyte migration to gut-associated lymphoid tissue (GALT) and persistently suppresses adaptive immune-mediated IBD [505-507]. Additionally, integrin αvβ5 is highly expressed on mature intestinal macrophages but not other immune cells in the mouse intestine, acts as a receptor for apoptotic cell uptake and promotes tissue repair by regulating the homeostatic properties of intestinal macrophages, such as angiogenesis and ECM remodelling [64]. Integrin αvβ6 is expressed only in epithelial cells and is mainly regulated by the integrin β6 (ITGB6) gene, which can increase integrin ligand expression, macrophage infiltration, proinflammatory cytokine secretion, and signal transducer and activator of transcription 1 (STAT1) signaling pathway activation. ITGB6 transgenic mice were found to have increased susceptibility to both acute and chronic dextran sulfate sodium-induced colitis, and αvβ6 induces intestinal fibrosis through the FAK/AKT pathway [508,509].

Anti-inflammatory treatment is ineffective in the development of fibrosis in IBD, a consequence of chronic inflammation. The mechanism of fibrosis is thought to be a continuous interaction between the stiffened ECM matrix resulting from the aberrant release of ECM components and cellular compartments [510]. During tissue injury, matrix deposition and turnover are highly disrupted, resulting in dysregulated matrix stiffness in the ECM [511,512]. Increased matrix stiffness triggers colonic myofibroblast activation to produce a fibrogenic phenotype and autopropagate fibrosis [513]. The expression of genes related to inflammatory and fibrogenic remodelling was significantly increased, suggesting the presence of both fibrosis and inflammation in CD strictures. Interstitial ECM is the most fundamental in the process of fibrosis, including the latent state of TGF-β, EGF, fibroblast growth factor (FGF) and other molecular fibrotic mediators [514]. αv and β5 are the major integrin isoforms in intestinal fibrosis, and their main function is to activate TGF-β. αvβ8 binds to a linear RGD motif of latent TGF-β, which subsequently recruits MMP14 and then releases TGF-β through proteolytic cleavage. αvβ8 can also activate TGF-β independently from cytoskeletal forces without release from latent peptide [256]. *In vivo* studies have shown that overexpression of αvβ6 in the epidermis activates TGF-β1, resulting in chronic ulcers and fibrosis [515]. Latent TGF-β1 was also activated through integrin αvβ3 expressed in human and rat intestinal smooth muscles [516], leading to the production of collagen I and fibrosis in CD [517]. The

elevated expression of α3β1 can enhance the expression level of MMP9 in keratinocytes through the TGF-β pathway [518].

Natalizumab (anti-α4 antibody) and vedolizumab (anti-α4β7 antibody) have been approved for maintaining clinical remission in patients with IBD [519,520]. Natalizumab was the first drug approved for the treatment of Crohn's disease, but its use has been limited because of its risk of progressive multifocal leukoencephalopathy [521,522]. Compared with natalizumab, vedolizumab acts specifically on α4β7 to selectively inhibit the trafficking of lymphocytes in the intestine. It has been approved for the treatment of IBD with few systemic adverse effects [523,524]. Currently, several anti-integrin drugs are undergoing more clinical trials. Abrilumab, a fully human monoclonal IgG2 antibody against the α4β7 integrin heterodimer, shows encouraging results in two phase II studies on moderate to severe CD and UC (CD: NCT01696396, UC: NCT01694485) [525,526], while no phase III clinical trial registration information has been found to date. Etrolizumab is a monoclonal antibody that specifically targets the β7 subunit of α4β7 and αEβ7 integrins to block their interaction with MAdCAM-1 and E-cadherin, respectively, which is in an ongoing robust phase II study on UC and a phase III study on CD. Notably, a phase I study of etrolizumab to evaluate its pharmacokinetics, pharmacodynamics and safety in pediatric patients 4 to <18 years of age with moderate to severe ulcerative colitis (UC) or with moderate to severe CD has been registered. AJM300, an oral α4 integrin antagonist characterized by mild adverse effects sharing a similar mechanism with natalizumab [527,528], is currently in a phase III study of patients with active UC (NCT03531892).

Multiple sclerosis (MS). MS is an autoimmune disease driven by agnogenic chronic inflammation in the central nervous system (CNS). It is characterized by inflammation in the brain and spinal cord that causes the demyelination of neurons, which blocks nerve signal transmission [529]. MS patients show sensory disorders, motor dysfunction, optic neuritis, and other physical and cognitive disorders [529]. Currently, there are approximately 2.5 million people with MS worldwide [530], which is a huge burden to society. The infiltration of autoreactive immune cells from peripheral circulation into the brain is the core pathogenesis of MS [531]. Preventing the infiltration processes of leukocytes into the CNS is an effective way to curb the progression of MS. Therefore, the adhesion molecules involved in leukocyte activation and mediating leukocyte migration to the CNS have received extensive attention. Among them, leukocyte integrins, as mentioned above, play important roles in regulating leukocyte function. In fact, in recent years, studies on the role of integrins in MS have yielded exciting results. In particular, integrin α4. Integrin α4 pairs with integrin β1, β2, or β7, of which integrin α4β1 is regarded as an important therapeutic target for MS. Integrin α4β1 is also called very late antigen-4 (VLA-4), which binds primarily to VCAM-1 and ECM ligand fibronectin deposited in inflamed tissues. The interaction between integrin α4β1 and VCAM-1 promotes the homing of leukocytes into the CNS, which accelerates the progression of MS. Disturbing the interaction between integrin α4β1 and VCAM-1 has been shown to effectively retard the progression of MS. As early as 1992, Yednok et al. demonstrated that inhibiting integrin α4β1 could effectively suppress the accumulation of leukocytes in the CNS, and they recommended anti-integrin α4β1 antibody as therapeutic for MS [532]. Natalizumab, a humanized IgG4 antibody that recognizes integrin α4, has been confirmed to significantly reduce the risk of the sustained progression of disability and the rate of clinical relapse in patients with relapsing MS. It could also enhance the therapeutic effect of interferon-β 1α (IFN-β 1α) on MS when combined with it. However, it has been reported that long-term use of natalizumab may cause serious infection

complications, such as progressive multiple leikoencephalitis (PML). Therefore, there is still a long way to go for the treatment of MS by targeting integrin α4β1. Novel integrin α4β1 inhibitors may be the key to overcoming MS in the future.

Rheumatoid arthritis(RA). RA is a chronic and systemic autoimmune inflammatory disease that is characterized by synovial hyperplasia, articular inflammation and synovial invasion into adjacent cartilage [533]. Integrins play an important role in the pathophysiology of RA, such as promoting communication between ECM proteins and rheumatoid cells and facilitating angiogenesis. αvβ3 and α5β1 are expressed on synoviocytes, including chondrocytes, fibroblasts and endothelial cells, and synovial-infiltrated cells, including T cells, neutrophils, B cells and macrophages, which promote binding to cartilage-pannus junctions and fibroblast invasion [534-536]. Fibronectin upregulated in inflamed articular tissues is a ligand of αvβ3 and α5β1 [534]. α5β1 promotes the proliferation of naive T cells and memory T cells by binding to fibronectin [534]. In RA, osteoclasts express αvβ3 at high levels, and αvβ3 promotes bone resorption because of osteoclast migration by recruiting c-Src kinase [537]. Macrophages and Th cells expressing αvβ3 and α5β1 produce IL-17, IL-1 and tumor necrosis factor (TNF)-α, which lead to the activation of synovial fibroblasts [538,539]. Neutrophils express αvβ3 and α5β1, which contribute to neutrophil migration and mediate cell adhesion to neutrophil extracellular traps (NETs) [536]. αvβ3 expressed by Th17 cells enables them to adhere to osteopontin, which serves as a costimulator of IL-17 [540]. Inhibition of αvβ3 prevents osteoclast-mediated bone destruction by reducing Th17 activation and receptor activator of nuclear factor-kappa B ligand (RANKL) levels [540]. In addition, integrins in RA could promote new vascularization. accumulation of synovial cells, and the secretions lead to hypoxia-inducible factor 1 (HIF-1) release, which acts as a stimulator of VEGF, PDGF and fibroblast growth factor 2 (FGF-2). These growth factors induced overexpression of αvβ3 and α5β1 in smooth muscle cells, endothelial cells and platelets. Upregulated αvβ3 and α5β1, in turn, further activate proinflammatory cytokine production, which mediates smooth muscle cell and endothelial cell proliferation and migration and platelet activation [541-543]. Furthermore, α9 is reported to be overexpressed both in animal models of arthritis and in RA patients, and increased α9 expression precedes the onset of arthritic symptoms. Blocking α9 inhibits fibroblast-like synoviocyte (FLS) activation against arthritis through a nonimmune-mediated mechanism [544].

In addition to the abovementioned diseases, integrins and their ligands are also involved in the progression of other autoimmune diseases. Multiple sclerosis is a demyelinating and inflammatory disorder of the CNS. Integrins such as α4β7, αEβ7, and α4β1 and their ligands are involved in the progression of multiple sclerosis by modulating the processes of immune cells [545]. B cells, neutrophils, and macrophages express high amounts of αMβ2, and systemic lupus erythematosus (SLE)-IgG enhances αMβ2-mediated adhesion to fibrinogen in systemic lupus erythematosus [546]. Inhibition of the α1β1 interaction with collagen leads to reduced accumulation of epidermal T cells, and the presence of anti-α6-integrin autoantibodies due to altered laminin integrity has been observed in psoriasis [547,548].

Integrin roles in other diseases

In addition to the above reports of integrin-related diseases, integrins also contribute to eye development and pathological processes, including the healing process of keratoconus injuries, allergic eye disease, cornea, lens opacification, diabetic retinopathy, glaucoma, eye infection, axon degeneration in the optic nerve and scleral remodelling in high myopia [549]. For example, α5β1 integrin participates in anchoring or integrating transplanted stem cells to the trabecular meshwork

in the eye for regeneration, and this might be a way for stem cell-based therapy for glaucoma [550]. Vitronectin/αv integrin-mediated NF-κB activation has been proven to induce inflammatory gene expression in bone marrow-derived macrophages. This will be an important step in the inflammatory process of dry eye disease (DED) [551]. In addition, drug discovery focused on integrin αlβ2, providing a marketed small molecule, LifiteGrast, for the topical treatment of DED [552]. For ophthalmic diseases, integrin inhibitors were proven to be effective in several preclinical models and have reported promising results in clinical trials [553].

Integrins are also promising antiresorptive therapeutic targets [554]. Osteoactivin promotes integrin β1 expression and leads to ERK activation. The expression of several genes upstream of osteoactivin was blocked, and the mRNA and protein levels of osteoactivin were decreased by dexamethasone. This ultimately inhibits integrin β1-ERK activation, resulting in reduced osteogenesis [555]. In addition, αvβ3 integrin participates in osteoclast differentiation and resorption, and αvβ3 integrin antagonists are considered to be effective drugs for postmenopausal osteoporosis [556]. L-000845704, as an αvβ3 integrin antagonist, was reported to inhibit bone resorption and improve bone mass in women with postmenopausal osteoporosis. A phase II clinical trial of 227 postmenopausal women with osteoporosis showed that L-000845704 could decrease the bone absorption marker carboxyterminal telopeptides of type I collagen (CTx) and increase the bone mineral density of the lumbar spine and femoral neck [557].

Alzheimer's disease (AD), characterized by cognitive decline, is a neurodegenerative disorder and is associated with amyloid-β (Aβ) plaque deposition, neuronal loss, and hyperphosphorylation of tau protein. Astrogliosis-associated AD is known to be caused by the interaction of amyloid β oligomers with β1-integrin. This enhanced β1-integrin and NADPH oxidase (NOX) 2 activity by NOX-dependent mechanisms [558]. In transgenic AD models, neutrophil depletion or inhibition of neutrophil trafficking by lymphocyte function-associated antigen (LFA)-1 blockade can reduce AD-like neuropathology and improve memory in mice showing cognitive dysfunction [559]. The counterligand of VCAM-1-α4β1 integrin, expressed by a large proportion of blood $CD8^+$ T cells and neutrophils, was abundant on circulating $CD4^+$ T cells in AD mice [560]. This suggested that α4 integrin-dependent leukocyte trafficking promoted cognitive impairment and AD neuropathology. Thus, the blockade of α4 integrins might be a new therapeutic method for AD. Recently, compared to isotype control injections without changing amyloid-β plaque load in a mouse model of AD, an antibody recognizing α4-integrin therapy reduced astrogliosis, microgliosis, and synaptic changes in APP/PS1 mice [561].

CHALLENGES AND OPPORTUNITIES: INTEGRIN-TARGETING DRUG DISCOVERY FROM BENCH TO CLINICAL

Integrins have historically been promising and challenging targets for the treatment of multiple diseases. The targeting integrin-related indications are summarized in Table 2, referring to cancer, fibrotic diseases, cardiovascular disease, viral infections, autoimmune diseases, and so on. The ongoing clinical studies of integrin-targeting drugs intended as disease therapies are summarized in Table 3 (from 2019 to 2022). Currently, there are approximately 90 kinds of integrin-targeting therapies in clinical trials, including integrin antagonists and imaging agents (search at https://www.clinicaltrials.gov, https://www.clinicaltrials-register.eu, https://www.australianclinicaltrials.gov.au, http://www.chictr.org.cn using the search term "integrin") (Table 4). Among them, approximately two-thirds of drugs or imaging agents are being studied in Phase I to Phase III, and nearly one-third of integrin-targeting therapies are terminated, withdrawn or no progression. The

related reasons are manifold, including delayed and difficult enrollment, lack of efficacy, safety concerns, commercial decision making and lack of funding. In 2022, the positive results in clinical trials show the new dawn of integrin-targeting therapies. For example, carotegrast (AJM300) is an oral, targeting α4-integrin small molecule antagonist, and the phase III study results showed that carotegrast was well tolerated and induced a clinical response in patients with moderately active ulcerative colitis who had an inadequate response or intolerance to mesalazine. Carotegrast, as the first oral anti-integrin drug, was approved by Japan's PMDA on March 28, 2022, for moderate ulcerative colitis (only when 5-aminosalicylic acid preparations are not adequately treated) [562]. Pliant Therapeutics, Inc. (PLRX) reported positive results for PLN-74809, the oral dual αvβ1/αvβ6 inhibitor, in the INTEGRIS-IPF Phase IIa study, which met its primary and secondary endpoints, demonstrating that PLN-74809 was well tolerated over the 12-week treatment period and showed a favorable pharmacokinetic profile. Herein, we summarize the main progression of small molecules, synthetic mimic peptides, antibodies, ADCs, peptide drug conjugates (PDCs), nanotherapeutic agents, CAR T-cell therapy, and imaging agents.

Small-molecule compounds and peptides

Small-molecule drugs accounted for the largest part of the ongoing clinical trials given their cost advantage, safety perspective, pharmacokinetic profiles, administration route, etc., compared with antibodies or larger conjugate molecules. Historically, many RGD-binding integrin drug discovery initiatives have been carried out to target the orthosteric binding sites, but most of these drug discoveries have not been successful due to the potential binding-induced conformational shifts of integrin from a low-affinity to a high-affinity state [28]. These reactions have been found for αIIbβ3 RGD mimetics such as eptifibatide and αvβ3 integrin RGD mimetics cilengitide, which shows direct agonist and proangiogenic effects at low doses.

In light of this potential effect, some research groups switched to identify non-RGD or pure small-molecule integrin antagonists and inhibitors binding allosterically. Another problem for drug discovery based on RGD-integrins is the undesirable physicochemical properties due to zwitterionic or amphoteric design. Therefore, novel chemotypes that are nonzwitterionic would be beneficial for oral bioavailability [28]. One of the first breakthroughs of non-RGD mimetics is RUC-1 and its more potent derivatives RUC-2 and RUC-4, targeting αIIbβ3 outside-in signaling pathways, which do not induce integrin activation [563,564]. A phase I, dose-escalation study showed that RUC-4 administered subcutaneously provided rapid, high-grade inhibition of platelet aggregation and that it is also safe and well tolerated and has the potential to be used at the point of first contact before primary coronary intervention [565]. RUC-4 was designed as a nonzwitterionic chemotype that does not potentially induce conformational shifts, which provides a promising approach for the discovery of αv-containing integrin antagonists. Other αvβ3 small-molecule pure antagonists, TDI-4161 and TDI-3761, have been designed and proven to not induce the conformational change tested by cryogenic electron microscopy imaging of integrin conformations [566]. Recent studies have shown that failed integrin small-molecule inhibitors in clinical trials are capable of stabilizing the extended open conformation with high affinity [49]. Closing inhibitors show a simple chemical feature with a polar nitrogen atom that stabilizes integrins in their bent-closed conformation by intervening between the serine residue and MIDAS [49].

The rational design of molecules that bind to integrin outside the ligand binding site, the allosteric site, could prevent integrin activation by sealing the orthosteric site or by keeping or promoting the conformation at a low-affinity state [28]. There are only reported some antibodies

targeting the allosteric site, such as natalizumab [567]. In recent years, novel chemotypes with high-quality orally bioavailable inhibitors have made large breakthroughs, such as carotegrast [562], PLN-74809 [568], and PTG-100 [569]. Although PTG-100, an oral α4β7 antagonist peptide, initially did not meet the primary endpoint in a phase IIa study, it showed proof-of-concept efficacy in patients with moderate-to-severe active UC, and the related data also suggested that local gut activity of an oral α4β7 inhibitor is important for efficacy for UC treatment, which is different from full-target engagement in blood. Other orally bioavailable inhibitors under ongoing clinical studies include IDL-2965 and MORF-057, developed by EA Pharma, Pliant, Protagonist, Indalo, and Morphic, respectively (Supplement Table S2).

Antibodies, ADCs and PDCs

Many monoclonal antibodies (mAbs) targeting integrins are now available as research tools or life-changing therapeutics and are classified into three groups: inhibitory mAbs acting as antagonists, stimulatory or activation-specific mAbs, and nonfunctional mAbs [570]. Anti-integrin mAbs are essentially competitive inhibitors, and most act as allosteric inhibitors, recognizing various parts of the ectodomain of subunit- or conformation-specific integrins [5]. Abciximab, an antibody against integrin αIIbβ3, has undergone extensive clinical studies (EPIC, EPILOG, CAPTURE) [571] and has been approved for use during PCI or in patients with unstable angina/non-ST-elevation myocardial infarction that did not respond to traditional treatment [84]. The integrin α4 antibody natalizumab has shown considerable therapeutic effects on multiple sclerosis [562]. Vedolizumab, an integrin α4β7 antibody, was used to treat Crohn's disease and ulcerative colitis [562]. Recently, abrilumab (Amgn), also called AMG-181, targeting the integrin α4β7 heterodimer, showed encouraging results in a phase II study on moderate to severe CD and UC [562]. AJM300 is an oral antagonist of integrin α4, which is currently in a phase III study of patients with active UC [562]. Integrin av mAbs have a range of selectivity profiles, which are beneficial in the validation of integrin targets in disease, but highly selective av small-molecule inhibitors are unavailable [572]. Currently, an example is P5H9 (MAB2528) for αvβ5 [573]. Currently, the antibody in the highest clinical trial stage is Etrolizumab, targeting integrin β7, which recently carried out a head-to-head comparison, phase III study, with infliximab, approved anti-TNF-α antibody, for the treatment of moderately to severely active ulcerative colitis (GAEDENIA) [574]. Overall, the GARDENIA study demonstrated that etrolizumab and infliximab achieved the same efficacy and safety endpoints at weeks 10 and 54 [575]. This head-to-head comparison also shows that the safety of the two in long-term results at one year is comparable.

Integrins, as cell surface receptors, are overexpressed in specific diseased tissues, which makes them design ADCs and PDCs to conjugate integrin binding antibodies and peptides to bioactive moieties. Indeed, recent clinical trials (NCT04389632) and (CTR20221496) have been initiated to investigate an ADC and PDC that selectively recognize β6 and αvβ3, respectively, to target solid tumors.

Nanotherapeutic agents

Integrins have been considered potential targets for cancer treatment for a long time, but there are no approved anticancer drugs targeting integrin. Nanotherapeutics approaches applied in targeting integrin therapies probably overcome the limitations of conventional therapies used in cancer treatment to achieve more precise, safer, and highly effective therapeutics. Integrins, overexpressed on the surface of cancer cells, are viewed as beneficial targets for the preferential delivery of genes or drugs into cancer cells [576]. The delivery of RGD-based peptides to integrin

receptors could be helpful for the binding and liberation of drugs in the tumor vasculature. The majority of nanoparticles (NPs) modified with RGD peptide and loaded with nucleotides or drugs have been developed in preclinical studies. For example, αvβ3 integrin-targeting NPs obtained by coupling RGD ligands to the surface of PEGylated chitosan-poly(ethylene imine) hybrids showed high gene silencing efficiency and facilitated efficient siRNA delivery [577]. The RGD motif was also used to connect to PEG-PLA and loaded with paclitaxel (PTX) and its derivative docetaxel (DTX) to avoid their disadvantages of low solubility and dose-limiting toxicity [578]. The cyclopeptide isoDGR is found in aged fibronectin, where it is formed by deamidation of Asn in an asparagine-glycine-arginine (NGR) site, which is a new αvβ3-binding motif with high affinity and does not induce integrin allostery and activation [579,580]. Therefore, in future studies, isoDGR-based nanotherapeutic agents have potential applications in cancer treatment.

CAR T-cell therapy

Integrins are also used in immunotherapy by conjugating to CAR T cells. Currently, there are two kinds of CAR T-cell therapies in clinical studies. OPC-415 targeting β7 and Marnetegragene autotemcel targeting β3 were developed by Otsuka and Pocket, respectively. The active conformer of integrin β7 served as a novel multiple myeloma (MM)-specific target, and MMG49, in the N-terminal region of the β7 chain, derived CAR showed good anti-MM effects without normal hematopoietic cell damage [27]. Currently, OPC-415 targeting β7 CAR T-cell therapy is in a phase II study. Integrin αvβ3- and αvβ6-CAR T cells also show therapeutic potential in solid tumors, such as melanoma, triple-negative breast cancer, and cholangiocarcinoma [581,582].

Imaging agent

Molecular imaging is an important part of precision medicine and plays an important role in the early diagnosis, staging, prognostic evaluation, individualized treatment and efficacy monitoring of major diseases such as cancers. 2-Deoxy-2-[18F]fluoro-d-glucose ([18F]FDG) positron emission tomography combined with low-dose computed tomography ([18F]FDG-PET/CT) is currently the gold standard for the clinical imaging diagnosis of various malignant tumors. However, in recent years, the development of clinical application of PET imaging has entered a bottleneck period, mainly due to the complex preparation of positron-electron drugs and the high imaging cost. Compared with PET technology, single photon emission computed tomography (SPECT) has lower equipment and drug costs, a higher clinical penetration rate and a better application foundation. However, the lack of effective imaging agents, such as 18F-FDG, limits the SPECT technology to play a greater role in tumor diagnosis and efficacy evaluation. Currently, SPECT imaging agents in the clinical phase mainly focus on integrin αvβ3 due to its overexpression on the surface of tumor neovascular endothelial cells and many tumor cells and the high affinity of polypeptides containing RGD sequences. Therefore, targeting αvβ3 SPECT imaging agents has been developed. 99mTc-3PRGD2 is the first broad-spectrum SPECT tracer developed by Peking University targeting integrin αvβ3 for detecting tumors, imaging angiogenesis, and evaluating tumor response to therapy [583]. The phase III study showed the good efficacy of 99mTc-3PRGD2 for the evaluation of lung cancer progression. αvβ6 integrin also serves as a promising target for cancer imaging. ^{18}F-FP-R$_0$1-MG-F$_2$ is an integrin αvβ6-specific PET imaging agent developed by Stanford University. The pilot-phase PET/CT study showed good safety and radiation dose performance in pancreatic cancer patients [584]. Except for pancreatic cancer, the potential indications include idiopathic pulmonary fibrosis (IPF), primary sclerosing cholangitis, and COVID-19 pneumonia.

CONCLUSIONS AND PERSPECTIVES

Decades of investigation into the biological functions of integrins have suggested that integrins exhibit roles in the regulation of many aspects of human health and disease, and their molecular mechanisms and signal transduction are also strikingly complex. Considering the width and feasibility of therapeutic options, targeting integrins is an important avenue to explore. In recent decades, targeting integrin drug discovery has continued to move forward with its twists and its turns. Many of the lessons learned from the past are also valuable to achieve a heavy bomb in this field. We give perspective from three aspects: basic research, clinical research, and translational research.

For basic research, research on integrins is quite mature but also a newly reawakened field. It is important to validate the function of integrin targets in clinically predictive disease models and analyze the expression landscape in a large-scale cohort in different diseases and states, which contributes to success in clinical trials. Notably, current studies of integrin-targeted strategies are focused not only on extracellular but also on intracellular targets that involve both inside-out and outside-in signaling pathways. Several adaptors are known to interact with the cytoplasmic tails of β-integrins, including Gα13, focal adhesion kinase, ILK, and Syk, Src family kinases. For example, Gα13 binds directly to the ExE motif in the cytoplasmic domain of the integrin β subunits, and this binding occurs only during early outside-in signaling. A myristoylated ExE motif peptide selectively inhibits outside-in signaling, platelet spreading and the second wave of platelet aggregation by selectively inhibiting Gα13-integrin interaction. This strategy to inhibit outside-in signaling not affect primary platelet adhesion and aggregation, but limit the size of a thrombus to prevent vessel occlusion[398,585]. 14-3-3ζ synergizes c-Src to β3-integrin, and forms the 14-3-3ζ–c-Src–integrin-β3 complex during platelet activation. Interference with the formation of complex by myristoylated-KEATSTF-fragment (KF7) and 3',4',7'-trihydroxyisoflavone (THO) is a strategy to selectively inhibit outside-in signaling without disrupting the ligand binding of integrins [586]. Targeting intracellular targets via outside-in signaling pathways may provide new sights for avoiding the formation of potentially undesired conformational states. Considering the substantial clinical failure in targeting integrin in the orthosteric binding sites due to activation of integrin signaling, identification of other allosteric sites is urgently needed to develop candidates that target integrin at other sites. Clearly, the conformational states shift exists in αvβ3 and αIIbβ3 induced by their inhibitors, but it is not clear to other RGD-binding integrins or leukocyte cell adhesion integrins, collagen binding integrins, laminin binding integrins. Crystallographic structural analysis would be helpful to reveal the conformational change mechanism. Considering the width and complexity of biological function and signaling within the integrin family, whereas only a small part of integrin biology is known, further research is required to explore the much unknown field.

For clinical research, targeting integrin therapeutics may have their greatest utility as combination therapies with other agents considering the potential function of integrin inhibition in overcoming acquired resistance to chemotherapy, radiotherapy, targeted therapy (including VEGFR inhibitors) or therapy targeting the immune microenvironment. Currently, due to the complexity of solid tumors, the combination therapy of anti-tumor drugs with different mechanisms or targets is the mainstream strategy in the clinic to improve anti-tumor efficacy and overcome or delay drug resistance. The identification of robust biomarkers and imaging technology applications are required to find patients with tumors whose progression is driven by integrin signaling or to measure specific integrin expression levels in the recruited subjects, which could guide the best clinical use of integrin inhibitors. In addition to focusing on efficacy of integrin antagonists, we should also pay special

attention to the adverse effects of integrin antagonists in clinical applications or clinical trials. For example, the oral αIIbβ3 antagonists were associated with increased mortality compared to intravenous administration [24]. One explanation could be that some of the drugs have agonist-like activity, which may trigger "outside-to-inside" signals within the receptor-cell membrane complex, affect receptor conformational status and competency, membrane fluidity, and calcium metabolism [587], and potentially activate GPIIb/IIIa receptor, maintain procoagulant activity and P-selectin expression [588,589]. Moreover, progressive multifocal leukoencephalopathy (PML), a rare but serious opportunistic infection of the central nervous system, is the most concerning adverse event of integrin antagonists. Currently approved α4 integrin antagonist, natalizumab, is at high risk of developing PML [590]. Efalizumab, an αLβ2 integrin antagonist previously approved for the treatment of plaque psoriasis [591,592], was also withdrawn from the market due to the incidence of PML [593]. A restricted risk management plan is necessary to help reduce the potential risk of PML in clinical practice and clinical trials [594]. For example, patients with any neurologic symptoms, immunocompromised conditions, or those receive concurrent immunosuppressive therapy or anti-TNFα antibodies should be precluded [527,594]. Therefore, these related adverse effects should be taken into consideration in ongoing clinical trials and systematic post-marketing surveillance will contribute to the success of translational research and drug discovery of targeting integrin therapeutics.

For translational research, developing small molecules with new chemotypes, high affinity and good pharmacokinetic profile for oral dosing is challenging but has a huge market. The identification of novel non-RGD or pure antagonist chemotypes via high-throughput screening and targeting integrin and ECM interactions are important drug discovery directions. In addition, given the multifaceted roles of integrins as signaling molecules, dual-target drug development and multi-indicative simultaneous development will improve the efficiency and success rate. Dual-target novel agents may overcome resistance compared with single-target drugs and often improve treatment outcomes and have more predictable pharmacokinetics profiles than combination therapies. The development of dual-target inhibitors has become an attractive research field for human cancer treatment and may provide synergistic anticancer effects. For example, integrins combined with other cell adhesion molecules, such as CD44 and dual-target inhibitors of tubulin and αv-integrin, for cancer treatment are an untapped research field. Currently, for cardiovascular diseases and ulcerative colitis treatment, anti-integrin therapeutics have been a major success. In the future, targeting integrin drug discovery is gradually going forward to unmet medical needs, such as IPF, NASH, aggressive or resistant malignancy, etc. Based on robust target validation, integrins will provide new significant opportunities for a variety of indications.

In summary, integrins play a crucial role in human health and disease due to their expression in multiple cell types and widespread involvement in cellular processes. Knowledge of integrins in various diseases is progressing, but the drug discovery process is less than satisfactory. We hope the progression in basic research, clinical research, and translational research will establish realizable access for developing effective drugs for unmet medical needs.

ACKNOWLEDGMENTS

This research was funded by National High Level Hospital Clinical Research Funding (Scientific and Technological Achievements Transformation Incubation Guidance Fund Project of Peking University First Hospital) (No.2022CX11, No. 2022RT01); National Key R&D Program of China (No. 2020YFC2008304); National Natural Science Foundation of China (No. 81973320 and No.

81903714). Thanks to Pharmacodia database for retrieving clinical trial data.

AUTHOR CONTRIBUTIONS

X.P. and Y.C. conceived and organized the manuscript. X.P., Q.X., X.H., Z.Q., H.Z., Z.L., and Y.G. wrote the manuscript, prepared the figures and contributed to the discussion. R.X. and N.Z. researched data and prepared table. All authors have read and approved the article.

ADDITIONAL INFORMATION

The authors declare no competing interests.

Figure Legends

Fig. 1. Timeline of the historical milestone for the study of integrin receptors and their main antagonists and agents in the past four decades.

Fig. 2. The primary structure and representative conformations of integrins. a. Organization of domains within the primary structures. b. Arrangement of domains within the representative 3D crystal structure of integrins. c. Conformational change of integrins: bent closed, extended closed, and extended open conformations.

Fig. 3. Classification, distribution, and ligands of integrins. The inner ring shows the 24 integrins that are composed of 17 α subunits and 8 β subunits. They are divided into four categories, namely, RGD-binding integrins, leukocyte cell adhesion integrins, collagen binding integrins, and laminin binding integrins, according to their distribution, ligand specificity, and functions. The middle ring shows the distribution of integrins in different cell types. The outer ring indicates the ligands bound by different types of integrins

Fig. 4. Schematic overview of integrin activation-associated signalling cascades. Integrin activation is regulated by multiple external signals, such as ECM, mechanotransduction or signaling from non-ECM ligands, including growth factor receptors, hormones, and small molecules, which is termed the "outside-in" mechanism. ECM or non-ECM ligand binding and force application results in integrin clustering and initiates downstream signaling to the actin cytoskeleton through recruited talin and vinculin, where actin can simultaneously pull on integrins and further in turn promote force generation. The "outside-in" mechanism then triggers various signaling cascades that ultimately result in cell survival, proliferation, cell spreading and even tumorigenesis and metastasis. On the plasma membrane, there is also an "inside-out" mechanism, which regulates the displacement of intracellular integrin inhibitors and allows talin or kindlin binding to integrin β- tails, controlling integrin affinity for ECM components. For example, in neutrophils, both Talin-1 and Kindlin-3 are rapidly recruited to activate $\beta 2$ integrins induced by extracellular chemokines binding to GPCR (G-protein coupled receptor). Solid arrows indicate activation, the dotted line indicates recruiting, and the solid blunt end arcs indicate inhibitory effects.

Fig. 5. The expression and function of major integrins and their related cancer types and metastatic sites. The expression of integrins can vary considerably between normal and tumor tissue and is also associated with cancer types and organotrophic metastasis.

Fig. 6. Roles of integrins in fibrosis processes in NASH, PH, and ADPKD. The lower part of the circle shows the role of integrins in liver fibrosis in NASH. In hepatic cells (HCs), activated integrin $\alpha 9\beta 1$ is endocytosed by hepatocytes and secreted in the form of extracellular vesicles (EVs), which are further captured by MoMFs. Captured integrin $\alpha 9\beta 1$ mediates MoMF adhesion to liver sinusoidal endothelial cells (LSECs) by binding to VCAM-1, which accelerates liver fibrosis. In HSCs, integrin $\alpha 8\beta 1$ promotes liver fibrosis by activating TGF-β. The binding of integrin $\alpha v\beta 3$ with OPN could promote laminin and α-SMA expression, which causes ECM accumulation and fibrosis

progression. Integrin αvβ5 also binds with OPN and enhances liver fibrosis, but the underlying mechanism still needs to be clarified. In CD4+ T cells, the adhesion between integrin α4β7 and HC expressing MAdCAM-1 recruits CD4+ T cells to the liver, which induces liver inflammation and fibrosis. The left part of the circle shows the role of integrins in intimal fibrosis in PH. In the progression of PH, integrin α1, α8, αv, β1, and β3 are upregulated, and α5 is downregulated in PASMCs. Integrin α1 increases and α5 decreases the concentration of Ca2+, promoting intimal fibrosis. The binding between integrin αvβ3 and OPN activates FAK signal transduction, which might be involved in the processes of vascular remodelling. The right part of the circle shows the role of integrins in renal fibrosis in ADPKD. Integrin αvβ3 expressed in renal tubular epithelial cells binds with periostin, activating TGF-β and promoting renal fibrosis. Binding between integrin αvβ3 and OPN is also involved in the renal fibrosis process, but the underlying mechanism is unclear. Renal tubular epithelial cells expressing integrin β1 enhance the expression of collagen, fibronectin, and α-SMA, which promote renal fibrosis.

Fig. 7. Main roles of integrins in the process of AS. Integrin signaling can affect multiple processes in AS, including endothelial dysfunction and activation, leukocyte homing to the plaque, smooth muscle cell migration, and thrombosis. In the process of endothelial cell activation, ox-LDL activates α5β1, induces the FAK/ERK/p90RSK pathway and promotes NF-κB signaling. Shear stress can activate αvβ3 and induce PAK activation by binding to fibronectin, thereby promoting NF-κB activation. Both ox-LDL and shear stress generated by blood flow mediate the increased expression of proinflammatory genes (ICAM-1 and VCAM-1) after integrin ligation. During the process of leukocyte homing to plaques, αxβ2 and α4β1 interact with VCAM-1 on the endothelial cell surface, and αxβ2 and αLβ2 interact with ICAM1 to promote leukocyte adhesion. Integrins α4β1, α9β1 and αvβ3 on the surface of monocytes interact with osteopontin, which is expressed in atherosclerotic plaques, to promote monocyte migration and survival. Integrin αDβ2 is upregulated during macrophage foam cell formation. During vascular smooth muscle cell migration, αvβ3 binding with fibronectin, osteopontin, etc., mediates FAK activity and drives migration. In the process of thrombosis, integrins α2β1 and aIIbβ3 on platelets are involved in platelet adhesion, activation, aggregation and thrombosis.

Table 1. Integrins expression involved with SARS-CoV-2 infection.

Table 2. The targeting integrin-related indications in clinical trials.

Table 3. Recent integrin-targeting drugs intended as disease therapies in ongoing clinical studies (2019-2022).

Table 4. Integrin-targeting therapies in clinical trials.

24% Overall Similarity

Top sources found in the following databases:

- 12% Internet database
- 22% Publications database
- Crossref database
- Crossref Posted Content database
- 0% Submitted Works database

TOP SOURCES

The sources with the highest number of matches within the submission. Overlapping sources will not be displayed.

1	mdpi.com<br>Internet	1%
2	Yasaswi Gayatri Mishra, Bramanandam Manavathi. "Focal adhesion dy...<br>Crossref	1%
3	link.springer.com<br>Internet	1%
4	Aleksi Isomursu, Martina Lerche, Maria E. Taskinen, Johanna Ivaska, E...<br>Crossref	<1%
5	ncbi.nlm.nih.gov<br>Internet	<1%
6	science.gov<br>Internet	<1%
7	Yajun Zheng, Katerina Leftheris. "Insights into Protein-Ligand Interacti...<br>Crossref	<1%
8	frontiersin.org<br>Internet	<1%

9	atvb.ahajournals.org<br>Internet	<1%
10	Ahmad Khurshid, Ju Lee Eun, Shaikh Sibhghatulla, Kumar Anuj et al. "T...<br>Crossref	<1%
11	res.mdpi.com<br>Internet	<1%
12	nature.com<br>Internet	<1%
13	"Posters (Abstracts 289-2348)", Hepatology, 2019<br>Crossref	<1%
14	ashpublications.org<br>Internet	<1%
15	cell.com<br>Internet	<1%
16	Advances in Experimental Medicine and Biology, 2014.<br>Crossref	<1%
17	worldwidescience.org<br>Internet	<1%
18	Jihong Li, Yoshiyuki Fukase, Yi Shang, Wei Zou et al. "Novel Pure αVβ3 ...<br>Crossref	<1%
19	Yasmin A. Kadry, David A. Calderwood. "Chapter 22: Structural and sig...<br>Crossref	<1%
20	Payaningal R. Somanath. "Cooperation between integrin αvβ3 and VEG...<br>Crossref	<1%

21	Enrica Pietronigro, Elena Zenaro, Vittorina Della Bianca, Silvia Dusi et a...<br>Crossref	<1%
22	Hao Sun, Frederic Lagarrigue, Alexandre R. Gingras, Zhichao Fan, Klau...<br>Crossref	<1%
23	Masashi Yamada, Kiyotoshi Sekiguchi. "Molecular Basis of Laminin-Int...<br>Crossref	<1%
24	William J. Sandborn, Larry C. Mattheakis, Nishit B. Modi, David Pugatc...<br>Crossref	<1%
25	jcs.biologists.org<br>Internet	<1%
26	Alexandra C. Finney, Karen Y. Stokes, Christopher B. Pattillo, A. Wayne ...<br>Crossref	<1%
27	Hellyeh Hamidi, Johanna Ivaska. "Every step of the way: integrins in ca...<br>Crossref	<1%
28	Yuanjun Shen, Dmitry A. Goncharov, Theodore Avolio, Arnab Ray et al. "...<br>Crossref	<1%
29	"ASGCT 21st Annual Meeting Abstracts", Molecular Therapy, 2018<br>Crossref	<1%
30	Cédric Zeltz, Donald Gullberg. "The integrin-collagen connection - a glu...<br>Crossref	<1%
31	Ester Ballana, Eduardo Pauls, Bonaventura Clotet, Françoise Perron-Sie...<br>Crossref	<1%
32	G. F. GUIDETTI. "Integrin α₂β₁ induces phosp...<br>Crossref	<1%

33	Jonas Schnittert, Ruchi Bansal, Gert Storm, Jai Prakash. "Integrins in w...<br>Crossref	<1%
34	medworm.com<br>Internet	<1%
35	"Platelets in Thrombotic and Non-Thrombotic Disorders", Springer Scie...<br>Crossref	<1%
36	Jenny Z. Kechagia, Johanna Ivaska, Pere Roca-Cusachs. "Integrins as ...<br>Crossref	<1%
37	Richard J. D. Hatley, Simon J. F. Macdonald, Robert J. Slack, Joelle Le, ...<br>Crossref	<1%
38	english.cemcs.cas.cn<br>Internet	<1%
39	wjgnet.com<br>Internet	<1%
40	orcid.org<br>Internet	<1%
41	researchgate.net<br>Internet	<1%
42	Fu-Yang Lin, Jing Li, Yonghua Xie, Jianghai Zhu, Thi Thu Huong Nguye...<br>Crossref	<1%
43	Tu, Fei. "Roles of Endothelial Cell Heat Shock Protein A12B and β-Gluc...<br>Publication	<1%
44	Jiansong Huang, Xia Li, Xiaofeng Shi, Mark Zhu et al. "Platelet integrin ...<br>Crossref	<1%

45	Machiko Honda, Chiemi Kimura, Toshimitsu Uede, Shigeyuki Kon. "Neu...<br>Crossref	<1%
46	Shuang Liu. "Radiolabeled Cyclic RGD Peptide Bioconjugates as Radiot...<br>Crossref	<1%
47	clinicaltrials.gov<br>Internet	<1%
48	Encyclopedic Reference of Vascular Biology & Pathology, 2000.<br>Crossref	<1%
49	Pullamsetti, Soni, Friedrich Grimminger, and Ralph Schermuly. "Novel a...<br>Crossref	<1%
50	k-ris.keio.ac.jp<br>Internet	<1%
51	biospace.com<br>Internet	<1%
52	Francisco Merino-Casallo, Maria Jose Gomez-Benito, Silvia Hervas-Ral...<br>Crossref	<1%
53	digitalcommons.lsu.edu<br>Internet	<1%
54	Dean J. Kereiakes, Tim D. Henry, Anthony N. DeMaria, Ohad Bentur et a...<br>Crossref	<1%
55	Arman Jahangiri, Alan Nguyen, Ankush Chandra, Maxim K. Sidorov et a...<br>Crossref	<1%
56	Hengameh Shams, Mohammad R.K. Mofrad. "α-Actinin Induces a Kink ...<br>Crossref	<1%

57	Hengameh Shams, Mohammad Soheilypour, Mohaddeseh Peyro, Ruho...<br>Crossref	<1%
58	Ivana Bravatà, Mariangela Allocca, Gionata Fiorino, Silvio Danese. "Inte...<br>Crossref	<1%
59	bmcbiol.biomedcentral.com<br>Internet	<1%
60	mscare.org<br>Internet	<1%
61	Robert A. Civitarese, Andras Kapus, Christopher A. McCulloch, Kim A. ...<br>Crossref	<1%
62	Veronika Ramovs, Lisa te Molder, Arnoud Sonnenberg. "The opposing r...<br>Crossref	<1%
63	Yu Cheng, Yuanhui Ji. "RGD-modified polymer and liposome nanovehic...<br>Crossref	<1%
64	docksci.com<br>Internet	<1%
65	annualreviews.org<br>Internet	<1%
66	"Signaling Pathways in Liver Diseases", Springer Science and Business...<br>Crossref	<1%
67	Arwa Morshed, Abdul Baset Abbas, Jialiang Hu, Hanmei Xu. "Shedding ...<br>Crossref	<1%
68	Carsten Höltke. "isoDGR-Peptides for Integrin Targeting: Is the Time U...<br>Crossref	<1%

69	Dhaliwal, Dolly. "Investigation of β5 Integrin Function in Epithelial Ovari...<br>Publication	<1%
70	Martijn A. Nolte, Esther N.M. Nolte-'t Hoen, Coert Margadant. "Integrins...<br>Crossref	<1%
71	Siqi Xiong, Yi Xu, Yiwen Wang, Ajay Kumar, Donna M. Peters, Yiqin Du. ...<br>Crossref	<1%
72	"Metastasis of Breast Cancer", Springer Science and Business Media L...<br>Crossref	<1%
73	Chao-yue Su, Jing-quan Li, Ling-ling Zhang, Hui Wang et al. "The Biolog...<br>Crossref	<1%
74	Duygu Sari-Ak, Alvaro Torres-Gomez, Yavuz-Furkan Yazicioglu, Anthos...<br>Crossref	<1%
75	Huimin Lu, Tao Wang, Jing Li, Carmine Fedele et al. "αvβ6 Integrin Pro...<br>Crossref	<1%
76	Lixiang Chen, Ting Wang, Yaomei Wang, Jingxin Zhang et al. "Protein 4...<br>Crossref	<1%
77	Soham Chakraborty, Souradeep Banerjee, Manasven Raina, Shubhasis ...<br>Crossref	<1%
78	Valentina Garlatti, Sara Lovisa, Silvio Danese, Stefania Vetrano. "The M...<br>Crossref	<1%
79	Yi-Shen Zhu, Kexing Tang, Jiayi Lv. "Peptide-drug conjugate-based nov...<br>Crossref	<1%
80	Bryce LaFoya, Jordan Munroe, Alison Miyamoto, Michael Detweiler, Ja...<br>Crossref	<1%

81	M Luisetto, Edbey Khaled, GR Mashori, Ilman Ahnaf, AR Yesvi, OY Latys...<br>Crossref	<1%
82	circ.ahajournals.org<br>Internet	<1%
83	termedia.pl<br>Internet	<1%
84	"Eighth Meeting and the 10th Anniversary of the European Neurologica...<br>Crossref	<1%
85	Ane Wyssenbach, Tania Quintela, Francisco Llavero, Jose L. Zugaza, C...<br>Crossref	<1%
86	Birgit Leitinger. "Transmembrane Collagen Receptors", Annual Review ...<br>Crossref	<1%
87	Chang, Claire Wei-Ju. "The Role of G alpha 13 in Integrin-Dependent Ne...<br>Publication	<1%
88	Mahajan, Gautam. "Mechanobiology of Brain-Derived Cells During Dev...<br>Publication	<1%
89	Mayra Paolillo, Massimo Serra, Sergio Schinelli. "Integrins in glioblasto...<br>Crossref	<1%
90	Nektaria Makrilia, Anastasios Kollias, Leonidas Manolopoulos, Kostas ...<br>Crossref	<1%
91	Shiv Ram Krishn, Amrita Singh, Nicholas Bowler, Alexander N. Duffy et ...<br>Crossref	<1%
92	encyclopedia.pub<br>Internet	<1%

93	Donato Lacedonia, Michele Correale, Lucia Tricarico, Giulia Scioscia et ...<br>Crossref	<1%
94	P. Clezardin. "Integrins in Bone Metastasis Formation and Potential Th...<br>Crossref	<1%
95	Al-Jamal, R.. "Beta1 integrin in tissue remodelling and repair: From phe...<br>Crossref	<1%
96	Andrew E. Williams. "Immunology", Wiley, 2011<br>Crossref	<1%
97	Arjan van der Flier, Arnoud Sonnenberg. "Function and interactions of i...<br>Crossref	<1%
98	Harpal Jakhu, Gurveen Gill, Amarjot Singh. "Role of integrins in wound ...<br>Crossref	<1%
99	Minyang Fu, Dandan Peng, Tianxia Lan, Yuquan Wei, Xiawei Wei. "Multi...<br>Crossref	<1%
100	hal-amu.archives-ouvertes.fr<br>Internet	<1%
101	ideaexchange.uakron.edu<br>Internet	<1%
102	ira.lib.polyu.edu.hk<br>Internet	<1%
103	onlinelibrary.wiley.com<br>Internet	<1%
104	synapse.koreamed.org<br>Internet	<1%

105	jbc.org<br>Internet	<1%
106	semanticscholar.org<br>Internet	<1%
107	"The 20th Conference of the Asian Pacific Association for the Study of ...<br>Crossref	<1%
108	Brandon J. Beddingfield, Naoki Iwanaga, Prem P. Chapagain, Wenshu ...<br>Crossref	<1%
109	Haiying Li, Shuya Huang, Shengqing Wang, Li Wang, Lei Qi, Yun Zhang, ...<br>Crossref	<1%
110	Motomu Shimaoka. "Therapeutic antagonists and conformational regu...<br>Crossref	<1%
111	Olachi J. Mezu-Ndubuisi, Akhil Maheshwari. "The role of integrins in inf...<br>Crossref	<1%
112	Zhiqi Sun, Mercedes Costell, Reinhard Fässler. "Integrin activation by t...<br>Crossref	<1%
113	ictandhealth.com<br>Internet	<1%
114	"Posters", Journal of Thrombosis and Haemostasis, 07/2009<br>Crossref	<1%
115	Chunxia Su, Bo Su, Liang Tang, Yinmin Zhao, Caicun Zhou. "Effects of ...<br>Crossref	<1%
116	Johannes F Van Agthoven, Jian-Ping Xiong, José Luis Alonso, Xianlian...<br>Crossref	<1%

117	Melody G. Campbell, Anthony Cormier, Saburo Ito, Robert I. Seed et al. ...	Crossref	<1%
118	Nirav Dhanesha, Manasa K. Nayak, Prakash Doddapattar, Manish Jain, ...	Crossref	<1%
119	pubmed.ncbi.nlm.nih.gov	Internet	<1%
120	idexlab.com	Internet	<1%
121	G TARONE. "Integrin signalling: The tug-of-war in heart hypertrophy", C...	Crossref	<1%
122	Lihong Pan, Peiyuan Bai, Xinyu Weng, Jin Liu, Yingjie Chen, Siqin Chen, ...	Crossref	<1%
123	doaj.org	Internet	<1%
124	dokumen.pub	Internet	<1%
125	Giulia Martelli, Monica Baiula, Alberto Caligiana, Paola Galletti et al. "C...	Crossref	<1%
126	Joel Raborn, Wei Wang, Bing-Hao Luo. "Regulation of Integrin αIIbβ3 Li...	Crossref	<1%
127	Marta Ripamonti, Bernhard Wehrle-Haller, Ivan de Curtis. "Paxillin: A H...	Crossref	<1%
128	Thenappan Thenappan, Stephen Y. Chan, E. Kenneth Weir. "Role of extr...	Crossref	<1%

129	com-corp-prod2.cms.takeda.com<br>Internet	<1%
130	cshperspectives.cshlp.org<br>Internet	<1%
131	downloads.hindawi.com<br>Internet	<1%
132	Alexandra Laine, Ossama Labiad, Hector Hernandez-Vargas, Sebastien...<br>Crossref posted content	<1%
133	Federico Cacciapuoti. "Molecular mechanisms of left ventricular hyper...<br>Crossref	<1%
134	Jakobsson, Tomas. "Oxysterol Receptors LXRs and Coregulators in Ch...<br>Publication	<1%
135	Khurshid Ahmad, Eun Ju Lee, Sibhghatulla Shaikh, Anuj Kumar et al. "T...<br>Crossref	<1%
136	Lihong Pan, Peiyuan Bai, Xinyu Weng, Jin Liu, Yingjie Chen, Siqin Chen, ...<br>Crossref	<1%
137	Marialucia Gallorini, Simone Carradori. "Understanding collagen intera...<br>Crossref	<1%
138	Mechanosensitivity of the Heart, 2010.<br>Crossref	<1%
139	Sara Shojaei-Zarghani, Ahmad Yari Khosroushahi, Maryam Rafraf. "On...<br>Crossref	<1%
140	Sun, Cui-Cui, Xian-Jun Qu, and Zu-Hua Gao. "Integrins : players in canc...<br>Crossref	<1%

141	Xianchi Dong, Li-Zhi Mi, Jianghai Zhu, Wei Wang, Ping Hu, Bing-Hao Lu... Crossref	<1%
142	Yue Wang, Yuanyuan Zheng, Zuoyu Tu, Yongguo Dai, Hong Xu, Li Lv, Ji... Crossref	<1%
143	erj.ersjournals.com Internet	<1%
144	etheses.whiterose.ac.uk Internet	<1%
145	exonpublications.com Internet	<1%
146	hdl.handle.net Internet	<1%
147	pubs.asha.org Internet	<1%
148	spiral.imperial.ac.uk Internet	<1%
149	dianova.com Internet	<1%
150	ebi.ac.uk Internet	<1%
151	journaltocs.ac.uk Internet	<1%
152	"Full SNO 2020 Abstracts PDF", Neuro-Oncology, 2020 Crossref	<1%

153	Annette Scheid, Martin Meuli, Max Gassmann, Roland H. Wenger. "Gen...<br>Crossref	<1%
154	Bethany Powell Gray, Kathlynn C. Brown. "Combinatorial Peptide Librar...<br>Crossref	<1%
155	Francesca Nardelli, Cristina Paissoni, Giacomo Quilici, Alessandro Gori...<br>Crossref	<1%
156	Goult, B.T.. "The Structure of the N-Terminus of Kindlin-1: A Domain Im...<br>Crossref	<1%
157	Haas, Paige. "Systematic Investigation of Host-Pathogen Interactions I...<br>Publication	<1%
158	Kiyoshi Yoshimura, Kristen F. Meckel, Lindsay S. Laird, Christina Y. Chi...<br>Crossref	<1%
159	Manakan Betsy Srichai. "Integrin Structure and Function", Cell-Extracell...<br>Crossref	<1%
160	Ryan J. Leiphart, Dongning Chen, Ana P. Peredo, Abigail E. Loneker, Pa...<br>Crossref	<1%
161	Shimaoka, Motomu, Junichi Takagi, and Timothy A. Springer. "Confor...<br>Crossref	<1%
162	Stewart, P.L.. "Cell integrins: commonly used receptors for diverse vira...<br>Crossref	<1%
163	Zhu, Liang. "RAP1-triggered Pathways for Talin-mediated Integrin Activ...<br>Publication	<1%
164	drrajivdesaimd.com<br>Internet	<1%

165	edoc.ub.uni-muenchen.de<br>Internet	<1%
166	epdf.pub<br>Internet	<1%
167	era.ed.ac.uk<br>Internet	<1%
168	formation.e-cancer.fr<br>Internet	<1%
169	molecular-cancer.biomedcentral.com<br>Internet	<1%
170	pesquisa.bvsalud.org<br>Internet	<1%
171	qmro.qmul.ac.uk<br>Internet	<1%
172	amvis.cz<br>Internet	<1%
173	clinicaloptions.com<br>Internet	<1%
174	dovepress.com<br>Internet	<1%
175	e-sciencecentral.org<br>Internet	<1%
176	jcancer.org<br>Internet	<1%

177	karger.com<br>Internet	<1%
178	omicsdi.org<br>Internet	<1%
179	"Poster II", Multiple Sclerosis Journal, 2012.<br>Crossref	<1%
180	"WORLD TRANSPLANT CONGRESS 2006 POSTER ABSTRACTS", Ameri...<br>Crossref	<1%
181	Brian Estevez, Bo Shen, Xiaoping Du. "Targeting Integrin and Integrin Si...<br>Crossref	<1%
182	Fabienne Danhier, Aude Le Breton, Véronique Préat. "RGD-Based Strate...<br>Crossref	<1%
183	Gergana Gocheva, Anela Ivanova. "A Look at Receptor-Ligand Pairs for ...<br>Crossref	<1%
184	Gunjan Manocha, Atreyi Ghatak, Kendra Puig, Colin Combs. "Anti-α4β1 ...<br>Crossref	<1%
185	Helen M. Sheldrake, Laurence H. Patterson. "Strategies To Inhibit Tum...<br>Crossref	<1%
186	Katsuyoshi Matsuoka, Mamoru Watanabe, Toshihide Ohmori, Koichi N...<br>Crossref	<1%
187	Lanqing Cui, Yun Li, Yuan Lv, Feng Xue, Jian Liu. "Antimicrobial resista...<br>Crossref	<1%
188	Sofie Vandendriessche, Seppe Cambier, Paul Proost, Pedro E. Marques...<br>Crossref	<1%

189	Xiaokang Li, Lin Liu, Tian Li, Manling Liu, Yishi Wang, Heng Ma, Nan Mu...<br>Crossref	<1%
190	"Molecular Biology of the SARS-Coronavirus", Springer Science and Bu...<br>Crossref	<1%
191	Bing-Hao Luo, Timothy A. Springer. "Integrin structures and conformati...<br>Crossref	<1%
192	Derek P. Chew, Deepak L. Bhatt, Shelly Sapp, Eric J. Topol. "Increased ...<br>Crossref	<1%
193	Integrin-Ligand Interaction, 1997.<br>Crossref	<1%
194	James Arthos, Claudia Cicala, Fatima Nawaz, Siddappa N. Byrareddy e...<br>Crossref	<1%
195	Monica Baiula, Paola Galletti, Giulia Martelli, Roberto Soldati et al. "Ne...<br>Crossref	<1%
196	Nasreen Khalil. "TGF-β: from latent to active", Microbes and Infection, ...<br>Crossref	<1%
197	Shiv Ram Krishn, Amrita Singh, Nicholas Bowler, Alexander N. Duffy et ...<br>Crossref	<1%
198	Sung Chul Park, Yoon Tae Jeen. "Anti-integrin therapy for inflammator...<br>Crossref	<1%
